# BIN1 is a key regulator of proinflammatory and neurodegeneration-related activation in microglia

**DOI:** 10.1186/s13024-022-00535-x

**Published:** 2022-05-07

**Authors:** Ari Sudwarts, Supriya Ramesha, Tianwen Gao, Moorthi Ponnusamy, Shuai Wang, Mitchell Hansen, Alena Kozlova, Sara Bitarafan, Prateek Kumar, David Beaulieu-Abdelahad, Xiaolin Zhang, Lisa Collier, Charles Szekeres, Levi B. Wood, Jubao Duan, Gopal Thinakaran, Srikant Rangaraju

**Affiliations:** 1 Byrd Alzheimer’s Center and Research Institute, University of South Florida, Tampa, FL 33613 USA; 2 Department of Molecular Medicine, Morsani College of Medicine, University of South Florida, Tampa, FL 33620 USA; 3 Department of Neurology, Emory University, Atlanta, GA 30322 USA; 4 Center for Psychiatric Genetics, North Shore University Health System, Evanston, IL 60201 USA; 5 Parker H. Petit Institute for Bioengineering and Bioscience, Wallace H. Coulter Department of Biomedical Engineering, and Georgia W. Woodruff School of Mechanical Engineering, Georgia Institute of Technology, Atlanta, GA 30332 USA; 6 Department of Psychiatry and Behavioral Neuroscience, University of Chicago, Chicago, IL 60637 USA

**Keywords:** BIN1, Alzheimer’s disease, Neuroinflammation, Microglia, Innate immunity, GWAS risk factor, LPS, IRF1, IRF7, PU.1, IFITM3, CX3CR1

## Abstract

**Background:**

The *BIN1* locus contains the second-most significant genetic risk factor for late-onset Alzheimer’s disease. *BIN1* undergoes alternate splicing to generate tissue- and cell-type-specific BIN1 isoforms, which regulate membrane dynamics in a range of crucial cellular processes. Whilst the expression of BIN1 in the brain has been characterized in neurons and oligodendrocytes in detail, information regarding microglial BIN1 expression is mainly limited to large-scale transcriptomic and proteomic data. Notably, BIN1 protein expression and its functional roles in microglia, a cell type most relevant to Alzheimer’s disease, have not been examined in depth.

**Methods:**

Microglial BIN1 expression was analyzed by immunostaining mouse and human brain, as well as by immunoblot and RT-PCR assays of isolated microglia or human iPSC-derived microglial cells. *Bin1* expression was ablated by siRNA knockdown in primary microglial cultures in vitro and Cre-lox mediated conditional deletion in adult mouse brain microglia in vivo. Regulation of neuroinflammatory microglial signatures by BIN1 in vitro and in vivo was characterized using NanoString gene panels and flow cytometry methods. The transcriptome data was explored by in silico pathway analysis and validated by complementary molecular approaches.

**Results:**

Here, we characterized microglial BIN1 expression in vitro and in vivo and ascertained microglia expressed BIN1 isoforms. By silencing *Bin1* expression in primary microglial cultures, we demonstrate that BIN1 regulates the activation of proinflammatory and disease-associated responses in microglia as measured by gene expression and cytokine production. Our transcriptomic profiling revealed key homeostatic and lipopolysaccharide (LPS)-induced inflammatory response pathways, as well as transcription factors PU.1 and IRF1 that are regulated by BIN1. Microglia-specific *Bin1* conditional knockout in vivo revealed novel roles of BIN1 in regulating the expression of disease-associated genes while counteracting CX3CR1 signaling. The consensus from in vitro and in vivo findings showed that loss of *Bin1* impaired the ability of microglia to mount type 1 interferon responses to proinflammatory challenge, particularly the upregulation of a critical type 1 immune response gene, *Ifitm3*.

**Conclusions:**

Our convergent findings provide novel insights into microglial BIN1 function and demonstrate an essential role of microglial BIN1 in regulating brain inflammatory response and microglial phenotypic changes. Moreover, for the first time, our study shows a regulatory relationship between *Bin1* and *Ifitm3*, two Alzheimer’s disease-related genes in microglia. The requirement for BIN1 to regulate *Ifitm3* upregulation during inflammation has important implications for inflammatory responses during the pathogenesis and progression of many neurodegenerative diseases.

**Graphical Abstract:**

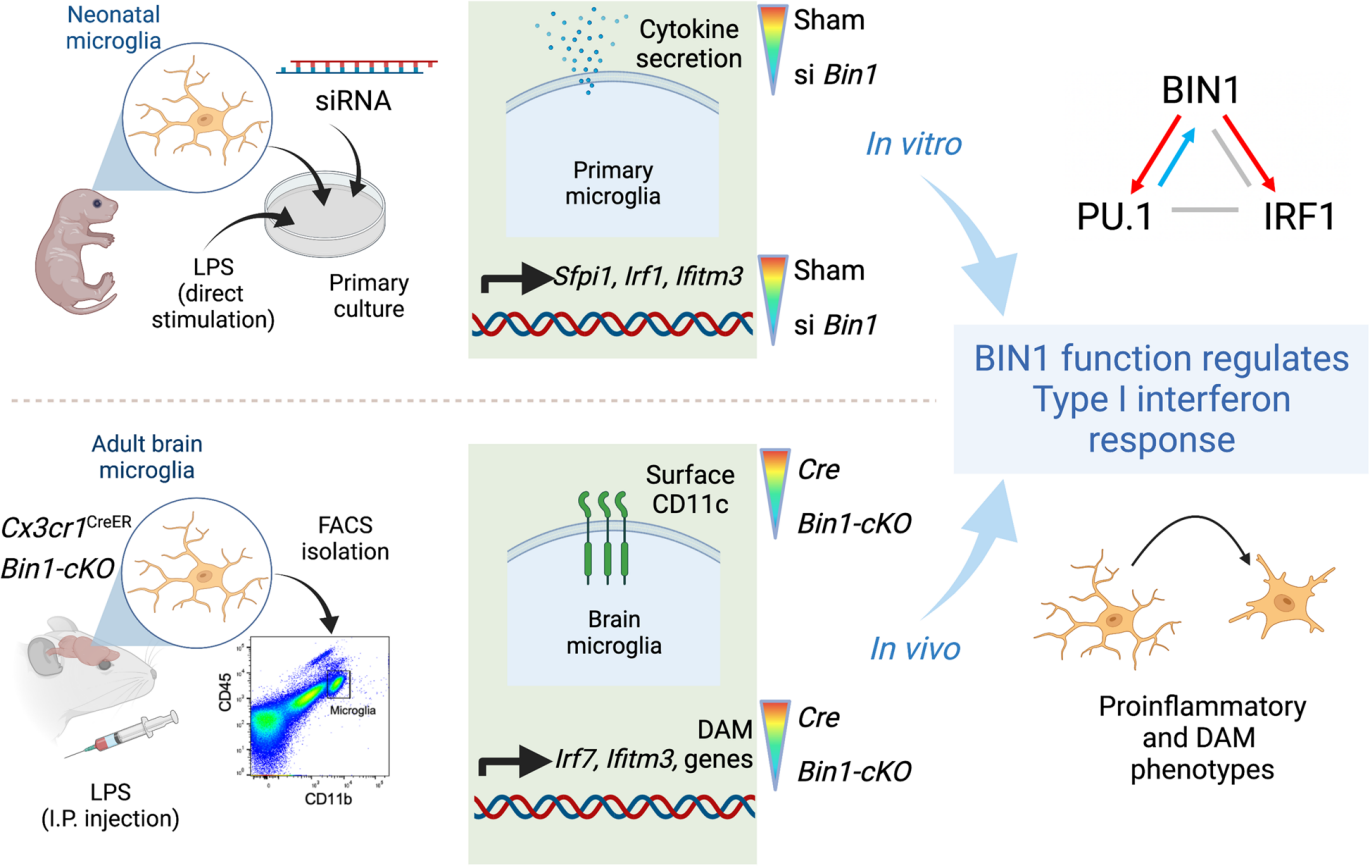

**Supplementary Information:**

The online version contains supplementary material available at 10.1186/s13024-022-00535-x.

## Background

Bridging Integrator 1 (*BIN1*) is a significant genetic risk factor locus for late-onset Alzheimer’s disease (LOAD) identified by genome-wide association studies [1–3]. Tissue- and cell-type-specific alternate splicing of seven out of the twenty *BIN1* exons generates multiple BIN1 isoforms, which vary in functional domains and differ in their subcellular localization [4]. BIN1 isoforms participate in a range of functions, including membrane remodeling, endocytosis, cytoskeleton regulation, and cell cycle [4]. Neuronal BIN1 localizes to presynaptic terminals in the mouse brain and plays an indispensable role in excitatory neurotransmission by regulating synaptic vesicle dynamics [5]. A central Clathrin-Associated Protein binding region (CLAP domain), present only in neuronal isoforms, confers BIN1’s ability to interact with the endocytic protein clathrin and its adaptor protein AP-2 [6]. Despite the importance of the endosomal pathway in β-amyloid production, the loss of neuronal BIN1 expression does not modulate β-amyloid pathology in a mouse model of Alzheimer’s disease (AD) amyloidosis [7]. Additionally, BIN1 can bind to tau and BIN1 overexpression induces tau-dependent network hyperexcitability in cultured neurons [8, 9], indicating that BIN1 may promote AD risk through tau pathogenesis. BIN1 has also been shown to limit the inter-neuronal spread of pathogenic tau in cultured neurons [10]. Moreover, independent studies have reported a significant decrease in neuronal BIN1 expression in individuals with AD [11, 12]. These latter findings are at odds with the suggestion that an increase in BIN1 expression contributes to tangle pathology. One possible explanation for this seeming inconsistency may be drawn from the observations that reduction in the neuronal isoform (iso 1) coincides with an increase in the ubiquitous isoform (iso 9) [11, 13].

In the CNS, ubiquitous BIN1 isoforms lacking the CLAP domain are expressed in neurons and non-neuronal cells, most prominently in oligodendrocytes within human brain white matter [11, 14, 15]. Interestingly, high-level *Bin1* transcript and protein expression have been reported in large-scale datasets of acutely isolated mouse and human brain microglia (Fig. S1) [16–18]. Moreover, in wild-type mice and mouse models of AD pathology, *Bin1* was in the top 20th percentile of abundant microglial proteins as assayed by quantitative mass spectrometry [19–21]. Still, it was found to be lower in abundance in phagocytic microglia isolated from mouse brain injected with apoptotic neurons [22], consistent with a potential homeostatic role of BIN1.

In human brain proteomic studies, Multi-marker Analysis of GenoMic Annotation (MAGMA) highlighted that protein co-expression modules enriched in microglia markers were also enriched in AD risk genes, implicating microglial dysfunction in LOAD pathogenesis [23]. Microglia are resident immune cells in the CNS with critical roles in brain development and function, including synapse pruning, neurogenesis, and immune surveillance of the brain [24]. Additionally, microglia play vital and complex disease-modifying roles in neuroinflammatory and neurodegenerative disorders, including AD, and transform from homeostatic states to disease-associated microglia (DAM) phenotypes through an immune checkpoint regulated by TREM2 [22, 25]. A recent network analysis of mouse microglial transcriptomic datasets also revealed heterogeneity within DAM phenotypes, namely proinflammatory and anti-inflammatory DAM sub-profiles [26]. In this framework of homeostatic microglia, pro- and anti-inflammatory DAM in AD, we found that *Bin1* has the highest module membership among AD-associated genes in a homeostatic gene module, raising the possibility that BIN1 may play functional and AD-relevant roles in microglia [21].

Phagocytosis and proliferation are hallmark microglial responses to AD-like pathologies [27, 28]. Despite the potential for BIN1 to regulate crucial cellular pathways and microglial functions under homeostatic and pathological conditions, studies to date have largely neglected to characterize microglial BIN1 expression. Seemingly, this oversight stems from unsuccessful efforts in previous investigations to visualize BIN1 immunoreactivity in microglia in an unambiguous manner. In this regard, high-level BIN1 expression in oligodendrocytes, myelin, and synapses results in the labeling of diverse cell populations; thus, slender BIN1-positive processes that densely overlap with each other are tightly packed throughout the depth of the histological sections [5, 11, 15]. BIN1 immunoreactivity in the relatively small microglial cells is therefore obscured by the more intense staining of oligodendrocyte and neuronal processes, posing technical challenges for a clear demonstration of microglial BIN1 protein expression in situ.

In light of myriad findings of microglial dysfunction and DAM transformation in AD pathogenesis [29, 30], it is critical to investigate microglial BIN1 expression and function. The present article achieves the first-layer analysis by documenting microglial BIN1 protein in the mouse brain and characterizing microglial BIN1 isoforms. Subsequently, we explored the functional role of BIN1 by silencing *Bin1* expression in primary mouse microglia and conditional knockout mice (cKO) via selective ablation of *Bin1* alleles in microglia. Utilizing neuroinflammatory transcriptomic profiling, we have identified BIN1 as a homeostatic microglial regulator that has a non-redundant role in the activation of proinflammatory responses upstream of *Apoe*, *Trem2,* and *Tyrobp,* and upstream of PU.1 and IRF1, both master regulators of microglial gene expression and transition to DAM [31, 32]. Loss of *Bin1* in vitro profoundly impaired microglial ability to respond to LPS, resulting in a blunted proinflammatory response as measured by cytokine production and gene expression. In vivo, loss of microglial *Bin1* in the systemic LPS-induced neuroinflammatory model also blunted proinflammatory gene expression changes in addition to diminishing the upregulation of several DAM genes. Consistent across in vitro and in vivo *Bin1* manipulation studies, BIN1 was predicted to regulate type 1 interferon response in microglia. Importantly, BIN1 was found to regulate inflammation-induced expression of *Ifitm3*, an interferon-response gene recently associated with AD-related mechanisms [33]. IFITM3 facilitates lysosome acidification [34, 35] and limits immune-response cytokine production [36, 37]. Notably, IFITM3 gene networks are enriched in brains and peripheral blood mononuclear cells of AD patients [38], suggesting that IFITM3 plays a role in microglial inflammatory responses to AD pathology. Collectively, these findings provide important insights into BIN1 expression and function in microglia, demonstrating the significance of microglial BIN1 expression in brain inflammatory response.

## Methods

### Animals, drug administration, and harvest

All experiments involving animals were conducted in accordance with the IACUC guidelines at the University of South Florida. *Bin1*^fl/fl^ strain was obtained from Dr. George C. Prendergast (Lankenau Institute for Medical Research) [39]. *Emx1*-IRES-*Cre* (JAX stock #005628) and *Cx3cr1*^*tm2.1(cre/ERT2)Litt*^/WganJ (JAX stock 021160; heterozygous mice are referred to as *Cx3cr1*^CreER^) lines were purchased from The Jackson Laboratory (Bar Harbor, ME). *Bin1*^fl/fl^ mice were crossed with *Emx1*-IRES-*Cre* or *Cx3cr1*^CreER^ animals to generate *Emx1*^Cre^:*Bin1*^fl/fl^ (*Emx*^Cre^-*Bin1* cKO) and *Cx3cr1*^CreER^:*Bin1*^fl/fl^ (*Cx3cr1*^CreER^-*Bin1* cKO) animals [5]. The mice were maintained on a C57BL6/J background.

Tamoxifen (10 mg/mL, prepared in a 10% ethanol and 90% sunflower seed oil solution by vortexing and sonicating) was administered through intraperitoneal injections (100 mg/kg) on 5 consecutive days. Mice were then rested for 4 weeks to allow the re-population of peripheral monocytes. Subsequently, LPS (dissolved in sterile saline at 250 μg/mL and filtered through a 0.22 μm syringe filter) was injected (750 μg/kg) on four consecutive days. The animals were sacrificed 24 h after the final injection. Mice were weighed prior to each LPS injection and monitored for sickness and weight loss. All animals were terminally anaesthetised with isoflurane overdose and perfused with ice-cold PBS. Brain tissue was dissected out and divided for microglial isolation and immunostaining.

### Immunofluorescence staining and imaging

Brain tissue was post-fixed at 4 °C for 24 h in PBS containing 4% paraformaldehyde and processed for paraffin embedding. Five μm-thick sections were deparaffinised in xylene, hydrated through an ethanol series, and subjected to antigen retrieval at 90 °C (in 10 mM sodium citrate containing 0.05% Tween 20, pH 6). After washing and permeabilization in 0.25% Triton X-100, non-specific binding sites were blocked by incubation at room temperature for 1 hour in a buffer containing 10% donkey serum, 3% BSA, and 0.1% Triton X-100 in TBS. The antibodies used for immunostaining are listed in Supplementary Table 5. Primary antibodies were diluted in 1% BSA (in TBS + 0.1% Triton X-100), added to slides, and incubated overnight at 4 °C. After washing, fluorescence-labeled secondary antibodies were added to slides and incubated for 2 h at room temperature. Alternatively, immunostaining was processed on an intelliPATH FLX automated staining system (Biocare Medical) following epitope retrieval and blocking non-specific binding sites according to the manufacturer’s instructions. The slides were sequentially incubated with each primary and secondary antibody for 1 h at room temperature. Sections were washed and dried at room temperature prior to mounting coverslips with VectorShield mounting medium. Images were acquired on an automated Nikon Eclipse Ti2 microscope fitted with the Yokogawa spinning disk field scanning confocal system and Photometrics PRIME 95B sCMOS camera, using 20X and 100X objectives. High magnification *z*-stack images were deconvolved in NIS-Elements software (Nikon), processed using Fiji/ImageJ, and converted into 2D projections by smooth manifold extraction plug-in [40].

### Fluorescence-activated microglial cell sorting

Mouse brain microglia were isolated by flow cytometry, as previously described [41]. Briefly, harvested brain tissue was minced on ice and passed through a 40 μm nylon cell strainer with ice-cold filtered PBS. The dispersed cell suspension was centrifuged at 800 x *g*, at 4 °C, for 5 mins, and pellet suspended in 35% isotonic Percoll solution containing 1x HBSS. Myelin was separated by centrifugation (800 x *g*, at 15 °C, for 25 mins) and removed from the top of the cell suspension. The Percoll solution was diluted > 10-fold in ice-cold PBS and centrifuged at 800 x *g*, at 4 °C, for 5 mins. The microglial cell pellet was resuspended in 300 μl ice-cold PBS. Dead cells were labeled with 7-aminoactinomycin D [1:1000] in PBS at room temperature for 30 mins. Cells were then labeled with APC-Cy7 rat α-CD11b[M1/70] (BD Pharmingen 557,657), PE-Cy7 rat α-mouse CD45[30-F11] (BD Pharmingen 552,848), and BV421 Armenian hamster α-CD11c[N418] (BD Horizon 565,452) [all diluted 1:100] in PBS for 30 mins, then washed twice with PBS. Flow cytometry was performed on a BD FACS Melody cell sorter. Live cells were gated by 7-aminoactinomycin D-negative staining. Mononuclear cells were gated by FSC-A/SSC-A and single cells by FSC-A/FSC-H. Microglial cells were isolated as CD11b^+^ and CD45^int^ population and collected in PBS and centrifuged at 1000 x *g* for 2 mins to sediment the cells. Cell pellets used for immunoblot analysis were snap-frozen on dry ice and stored at − 80 °C until further processing. Cell pellets used for NanoString analysis were immediately lysed in RLT buffer (Qiagen), snap-frozen on dry ice, and stored at − 80 °C.

### Primary neonatal microglia isolation

Primary mouse microglial cultures were established using established isolation and enrichment protocols [42, 43]. As described previously [31], C57BL/6 J mice (P0-3) were euthanized, and brains were dissected then digested with Trypsin for 15 min at 37 °C. After quenching the Trypsin with 20 ml DMEM (Dulbecco’s Modified Eagle Medium)/10% fetal bovine serum (FBS) and 1% penicillin-streptomycin-glutamine, the cellular pellet was washed, and myelin debris was removed. The remaining cell suspension was filtered through a 40 μm strainer followed by CD11b^+^ positive selection using the mini-MACS (Miltenyi Biotec Cat#130-042-201) column. CD11b^+^ enrichment resulted in > 90% pure CD11b^+^ microglia as previously validated by flow cytometry [42]. Cells were then seeded in poly-L-lysine-coated wells and cultured in DMEM. After 24 h, the medium was replaced with a fresh medium, after which cells were used for experimentation.

### Human iPSC-derived microglia-like cells

The generation of human iPSC lines from human blood cells and their characterization have been previously described [44, 45]. Human iPSCs were differentiated into primitive macrophage precursors and then to microglia (iMG) essentially as described [44, 46]. Final differentiation of primitive macrophage precursors into iMG occurred over 10 days, and cells were maintained in culture for at least 1 month before harvesting for immunoblot and RT-PCR analyses.

### Generation of *Bin1* KO BV2 pools

BV2 cells were cultured in DMEM supplemented with 10% fetal bovine serum, 4 mM L-glutamine, 100 U/ml penicillin, and 100 μg/ml streptomycin, at 37 °C and 5% CO_2_. Cells were transduced with lentiviruses generated using pLentiCRISPRv2 plasmids (Genscript) expressing *Bin1* sgRNA (GAAGGATCTTCGGACCTATC) or non-target sgRNA. Stably transduced pools were selected in puromycin, and *Bin1* deletion was assessed by PCR amplification across the gRNA target site (F-primer: ACTGAGTGGTGGCTGACAAG; R-primer: TGAGTGCCAGAGAATCAGCG) and sequencing. PCR products were also cloned into pGEM-T Easy (Promega), and four individual clones from each pool were sequenced to confirm deletions within the target region. WT and KO pools grown to 60 -70% confluency were serum-starved for 16 h and treated with LPS (0.5 μg/mL) for a further 16 h before harvesting for RNA isolation or lysate preparation.

### *Bin1* small interfering RNA (siRNA) transfection study

*Bin1* was silenced with *Bin1* siRNA (sc-29,805 Santa Cruz Biotechnology), and equal amounts of non-specific sham siRNA (sc-37,007) were used for control. Primary microglia were transfected with 40 nM (final concentration) of siRNA using Lipofectamine™ RNAiMAX (Invitrogen) and Opti-MEM (Invitrogen). After 48 h, the efficiency of siRNA-mediated gene silencing was confirmed by qRT-PCR. LPS (10 ng/ml or 100 ng/ml, Sigma-Aldrich Cat#L4391, *E. coli* 0111:B4) was added after 24 h of siRNA exposure to activate microglia. The cells were collected after 24 h of activation for qRT-PCR, NanoString, and phagocytosis studies, while supernatants were collected for cytokine assays. The viability of *Bin1* siRNA-treated cells was measured by flow cytometric assay by LIVE/DEAD Dye staining (Invitrogen) with heat-treated cells as a positive control.

### Immunoblot

Protein was extracted from whole-brain samples by homogenizing in lysis buffer (150 mM NaCl, 50 mM Tris-HCl, 0.5% NP-40, 0.5% SDS) supplemented with 1x Roche cOmplete protease inhibitors, 250 μM PMSF. DNA was sheared by sonication using a probe sonicator. Protein was extracted from microglia isolated by flow cytometry and cultured human iMG by trituration in lysis buffer. Aliquots of protein samples were electrophoresed through 4-20% Bis-Tris gels, and blots were probed with rabbit anti-BIN1 (Proteintech 14,647-1-AP, 1:1000) and mouse anti-β-actin (Proteintech 66,009-1-lg, 1:50,000) antibodies. The blots were developed with IR680- and IR800-conjugated secondary antibodies and imaged with the Odyssey Infrared Imaging System (Li-COR Biosciences).

### RT-PCR and isoform quantification

RNA was extracted from tissue and cells using the DirectZol kit (Zymo), per the manufacturer’s instructions. RNA was reverse transcribed using Superscript IV (Invitrogen), and PCRs were performed using Phusion polymerase (NEB). The PCR primers were designed to span exon 7 (6-F: GGATGAAGCCAAAATTGCCAA; 10-R: CATCATTGAGGTTCTGATTGAGC), the CLAP domain and exon 17 (12-F: AF690_CATCCCCAAGTCCCCATCTC; 19-R: AATCACCAACACCACATCGC), or exon 11 (10-F: TCAATGATGTCCTGGTCAGC; 12-R: GCTCATGGTTCACTCTGATC). Primers were also designed to amplify across the human exon 11 sequence (Hu_exon 10-F: AGAACCTCAATGATGTGCTGG; Hu_exon 12-R: TCGTGGTTGACTCTGATCTCGG). Amplified DNA fragments were electrophoresed through 7.5% acrylamide gels. The gels were stained using SYTO™ 60 dye (Invitrogen) and visualized on an infrared Odyssey scanner (LICOR). PCR products amplified using Alexa flour 690-modified forward primer were scanned without staining to allow semi-quantification of DNA based on fluorescence intensity relative to the molecular load.

**Fig. 1:**
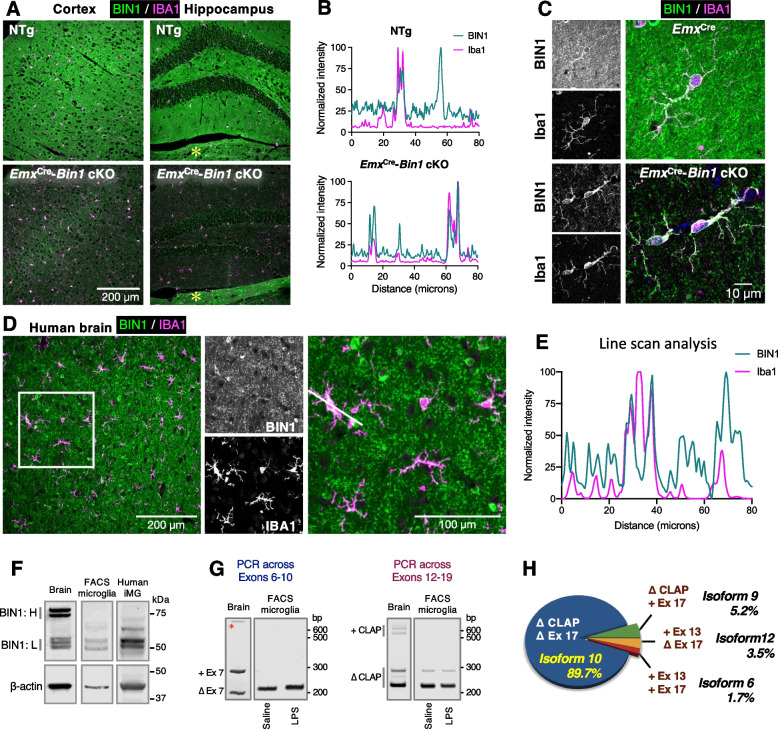
Characterization of BIN1 in the mouse brain and human iPSC-derived microglia. **A** Five μm-thick paraffin sections were stained with antibodies against BIN1 (green) and IBA1 (magenta). Images of the cortex and hippocampus from a WT animal show BIN1 expression in IBA1-positive microglia (top panel). By genetically ablating Bin1 expression from excitatory neurons and oligodendrocytes, microglial BIN1 expression is confirmed in Bin1 cKO mice (bottom panel). An asterisk indicates the expected unperturbed BIN1 expression in the thalamus beneath the dentate gyrus in the cKO brain [5]. **B** Line-scan analysis shows the concordance of BIN1 and IBA1 signal intensities in a subset of cells (in WT) and indicates the expression of BIN1 in IBA1+ microglia. The removal of BIN1 expression in excitatory neurons and oligodendrocytes demonstrates that the high-intensity profile of the microglial marker (IBA1) overlaps with that of BIN1, affirming microglial BIN1 expression. **C** Higher magnification images evidence BIN1 localization in the perinuclear regions of the microglial soma in Bin1 cKO and Emx^Cre^ littermates. Microglial BIN1 localization is readily apparent in the Bin1 cKO mouse brain, where BIN1 immunoreactivity could be seen permeating into cells’ ramifications (bottom panels). **D** Human post-mortem brain sections were stained with antibodies against BIN1 (green) and IBA1 (magenta). Overlapping morphological homogeneity of immunofluorescence unambiguously demonstrates BIN1 expression in human microglia. **E** Line-scan analysis exemplifies the overlapping expression of the two channels in D. Peaks in both channels represent microglial BIN1 expression. BIN1 only peaks reflect signals from oligodendrocyte cell bodies. The single isolated IBA1 peak suggests a lack of BIN1 expression in the nucleus of microglia. **F** Immunoblot analysis of BIN1 expression in whole-brain homogenates shows higher levels of BIN1 isoforms containing the CLAP domain (BIN1: H) and lower levels of BIN1:L isoforms. In contrast, FACS-isolated mouse microglia and human iPSC-derived iMG predominantly express BIN1 isoforms lacking the CLAP domain (BIN1: L). **G** RT-PCR analyses of FACS-isolated microglia demonstrate that exon 7 (left) and the CLAP domain (right) are excluded in the majority of microglial Bin1 transcripts. We detected no relative change in Bin1 isoforms following LPS administration (see Figs. S2A and S5H). An asterisk indicates a non-specific PCR product. **H** Microglial Bin1 isoforms generated by alternative splicing. Cloning and individual analysis of the PCR products allowed the Bin1 isoform frequency to be calculated. Approximately 90% of mouse microglial Bin1 transcripts code for isoform 10, with isoforms 9, 12, and 6 together, accounting for approximately 10% of Bin1 transcripts. Exon 7 (within the BAR domain), exon 11 (PI domain; see Fig. S3A), and exons 14-16 (within the CLAP domain) were not present in any microglial isoforms screened

For isoform frequency calculation, FACS-isolated microglial cDNA was used for PCR amplification using primers 12-F and 19-R. The products were purified and cloned in pGEM-T Easy vector (Promega) and transformed into JM109 cells. DNA isolated from individual colonies was re-amplified by PCR and electrophoresed through 5% acrylamide TBE gels to distinguish splicing by the insert length. All larger inserts and a selection of the most frequent (and readily distinguishable, smallest) inserts were analyzed by sequencing to identify the four isoforms expressed in mouse brain microglia (Fig. S2B). The relative frequency of clones corresponding to each of the four isoforms was calculated, and the data are presented in Fig. 1H.

### Quantitative reverse transcriptase PCR (qRT-PCR)

RNA extraction and cDNA synthesis were performed as described previously [47]. Quantitative real-time PCR was performed on 7500 Fast Real-time PCR System using TaqMan PCR master mix (Applied Biosystems). The following gene-specific TaqMan probes were used: *Trem2* (Mm04209424_g1), *Apoe* (Mm01307193_g1), *Tyrobp* (Mm00449152_m1), *Spp1* (Mm00436767_m1), *Grn* (Mm00433848_m1), *Lamp1* (Mm00495262_m1), *Bin1* (Mm00437457_m1), and *Gapdh* (Mm99999915_g1). Each sample was analyzed in duplicates, and the relative gene expression analysis was calculated using the 2^-ΔΔCt^ method compared to the housekeeping gene *Gapdh* [31]. Alternatively, RNA extraction and cDNA synthesis were conducted in the same manner described for RT-PCR. qRT-PCR was then conducted using a QuantStudio™3 Real-Time PCR System (Applied Biosystems). Samples were amplified using technical triplicates (using primer sequences listed in Supplementary Table 6). Relative gene expression analysis was calculated using the 2^-∆∆Ct^ method, normalised to *Cotl1*.

### Quantitative NanoString neuroinflammatory gene expression and data analysis

Microglia were exposed to siRNAs against *Bin1* for 24 h, followed by LPS treatment (10 ng/ml) for an additional 24 h, after which cells were lysed in TRIzol (Invitrogen). RNA was then isolated using the nCounter low RNA input kit (NanoString LOW-RNA-48). Quality control checks were performed on all samples to determine RNA concentration and integrity (RIN scores > 8.8 for all samples), and 50 ng of each sample was used for the NanoString assay using the NanoString Neuroinflammation panel (770 selected genes) [48]. Gene expression was measured using the NanoString, and the genes with counts two standard deviations above the negative control geomean were included in the final analysis. Of 770 genes represented in the panel, 681 genes met this criterion. The counts per gene were then normalized to the geometric mean of 8 housekeeping genes included in the panel. Principal component analysis (PCA) of the expression dataset was first performed to determine whether experimental conditions clustered together and to identify the Bin1 and LPS effect on the dataset. K-means cluster analysis was performed using Morpheus software (Broad Institute). As an orthogonal clustering approach, tSNE was also performed on the NanoString expression data. The agreement between K-means and tSNE clusters was determined by overlaying the two-dimensional tSNE scatter plot with K-means cluster membership (SPSS Version 24). Group-wise analysis of variance (ANOVA) followed by post-hoc Tukey’s test was performed for comparisons across groups. Gene ontology (GO) analysis was performed to identify enriched GO terms, Wikipathways, and KEGG pathways within each cluster using all 681 included genes as the reference list (GOElite, Version 1.2.5) [48, 49].

### Fluorescent polystyrene microsphere phagocytosis flow Cytometric assay

Phycoerythrin (PE)-conjugated polystyrene microspheres (Thermo-Fisher Fluorospheres, Cat Cat#F13083) were added to primary microglia [41, 50]. Cells were exposed to 5 μl microspheres (≈200 microspheres/cell) for 1 h at 37 °C followed by trypsin incubation for 10 min at 37 °C to detach the cells, after which DMEM with 10% fetal bovine serum was added. The cells were harvested while on ice to halt phagocytic activity. The cells were washed with ice-cold PBS and then labeled with fluorophore-conjugated CD45 (CD45-BV421, BD Biosciences Cat#563890) for 30 min at room temperature, followed by washing prior to flow cytometry. Phagocytic characteristics were assayed by flow cytometry as previously described [50]. All flow cytometric data were analyzed using FlowJo version 10, and proportions of cells demonstrating phagocytic uptake of > 1 bead/cell were determined as an index of phagocytic activity. Phagocytic uptake of > 2 beads/cell was regarded as high-level phagocytosis.

Phagocytosis was assessed in adult mouse brain cells (containing unpurified microglia) following processing in the same manner described for fluorescence-activated microglial cell sorting. Following myelin removal, cells were incubated with 1 μl yellow-green polystyrene beads (Sigma, Cat#L4655) in 100 μl PBS and incubated in a humidified incubator at 37 °C with 5% CO_2_ for 1 h. Cells were then washed twice with PBS, stained with APC-Cy7 rat α-CD11b and PE-Cy7 rat α-CD45, and flow cytometry gated as described for fluorescence-activated microglial cell sorting.

### Fluorescent Fibrillar Aβ42 phagocytosis flow Cytometric assay

Fibrillar fluorescent Aβ42 conjugated to HiLyte Fluor 488 (fAβ42-488) was prepared by mixing 100 μg of peptide (Anaspec Cat#AS-60479) in 20 μl 1% NH_4_OH and immediately diluted with 1XPBS to prepare a 100 μM stock. The mixture was incubated at room temperature for 6 days and then used for phagocytosis assays as described previously [41, 50]. After in-vitro exposure to siRNA and/or inflammatory stimuli, fAβ42-488 (2 μM final concentration) was added for 1 h at 37 °C. Cells were harvested as discussed above, and then the washed cells were labeled with fluorophore-conjugated anti-CD45 mAb (CD45-PE-Cy7, BD Biosciences Cat#552848). Compensation experiments were performed using compensation beads. Phagocytic uptake of fluorescent fAβ42-488 within live CD45^+^ microglia was measured as a proportion of fluorescent cells. We have already previously shown that this peak of fluorescence is inhibited by cytochalasin D treatment, confirming that our assay measures actin-dependent phagocytic processes [50].

### Multiplex immunoassays of cytokines and chemokines (Meso scale discovery platform V-PLEX)

Culture supernatants were collected prior to harvesting cells for transcriptomic studies. Supernatants were centrifuged to remove debris and then 120 μl was used for multiplex immunoassays (MSD V-PLEX Proinflammatory panel: IFN-γ, IL-10, IL-12p70, IL-1β, IL-2, IL-4, IL-5, IL-6, KC/GRO, TNF-α), per manufacturer’s instructions. These experiments were performed at the Emory Multiplexed Immunoassay Core (EMIC), and all samples were run in duplicate. Standard curves were created for each cytokine. Cytokine data were normalized to the overall mean and represented as a heat map (Morpheus software, Broad Institute). Group-wise ANOVA and post-hoc pairwise statistical comparisons were performed. The same samples were also assayed using a Luminex cytokine panel (EMD Millipore, 15-plex cytokine kit: GM-CSF, IFN-γ, IL-10, IL-1α, IL-2, IL-4, IL-6, IP-10/CXCL10, MCP-1/CCL2, MIP-1α/CCL3, TNFα, M-CSF, VEGF-A, G-CSF, RANTES for in vitro studies). We also measured levels of 32 cytokines (EMD Millipore, 32-plex cytokine kit: G-CSF, Eotaxin, GM-CSF, IFN-g, IL-1a, IL-1b, IL-2, IL-4, IL-3, IL-5, IL-6, IL-7, IL-9, IL-10, IL-12p40, IL-12p70, LIF, IL-13, LIX, IL-15, IL-17, IP-10, KC, MCP-1, MIP-1a, MIP-1b, M-CSF, MIP-2, MIG, RANTES, VEGF, TNF-a, for in vivo studies). These assays were performed per the manufacturer’s protocols and read out on a MAGPIX instrument.

### Microglial morphology analysis

Mouse brains were post-fixed with 4% PFA at 4 °C, then equilibrated in 30% sucrose until the brains sank. Frozen sections (25 μm) were stained with goat α-IBA1 antibody (Novus Biologicals) for 40 h at 4 °C. Secondary antibody (Alexa fluor 555 donkey α-goat, Invitrogen) was incubated for 3 h, and nuclei were stained with Hoechst for 30 mins. Micrographs of whole-brain sections were acquired as 4.5 μm-thick *z*-stacks (at 0.5 μm intervals) using a Nikon Eclipse Ti2 microscope with a 20X objective. Image tiles were stitched together and deconvolved using NIS Elements software (Nikon). Maximum intensity projections of the stacked images were generated and converted into binary masks in Fiji/ImageJ. Individual IBA1^+^ cells within (specific regions primary somatosensory cortex, CA1, and hypothalamus) were selected as regions of interest. Morphometric analysis was conducted in Fiji/ImageJ using the FracLac plugin’s region of interest scan function [51]. Output images were inspected manually (by a blinded researcher) to ensure that convex hull detection by FracLac resembled the original maximum intensity projections. Hull and circle morphometric data were analysed with SPSS software.

### Statistical analyses

GraphPad Prism version 8.0, Microsoft Excel version 2017, SPSS version 24, and R (version 3.5.1) were used for data analyses and data representation. Data are shown as mean ± standard error of the mean (SEM). Student’s t-test (two-tailed, assuming equal variance) was used for pairwise comparisons, with statistical significance set at *p* < 0.05 unless otherwise specified. All other statistical considerations are discussed in the relevant sections above.

## Results

### BIN1 expression and subcellular localization in mouse and human brain microglia

Initially, we sought to unambiguously identify BIN1 protein expression in microglia in the mouse brain. We immunostained wild-type (WT) mouse brain sections with antibodies against BIN1 and IBA1 and found several cells positive for both proteins in the cortex and hippocampus (Fig. 1A, upper panel). However, the blanket of synaptic BIN1 throughout the brain parenchyma shrouded the entire field of view in the micrographs, producing poor contrast to identify cell specificity by morphology. In order to definitively confirm BIN1 expression in IBA1-positive microglial cells, we generated *Emx*^Cre^:*Bin1*^fl/fl^ mice in which *Bin1* alleles were deleted from excitatory neurons and oligodendrocytes of the hippocampi and cortices [5], providing better contrast for detection of BIN1 in unaffected cell populations (i.e., microglia). As expected, only low-level BIN1 immunoreactivity (likely in inhibitory synapses) was detected in the neuropil of the cortex and hippocampus of *Emx*^Cre^-*Bin1* cKO using a BIN1-specific antibody [5] (Fig. 1A, lower panel). In contrast, a typical BIN1 expression profile in oligodendrocytes and myelinated fiber tracts was evident in the midbrain (Fig. 1A, indicated by an *asterisk*). Unlike the cellular BIN1 immunofluorescence staining in both IBA1^+^ and IBA1^−^ cells in WT mice, BIN1 cellular staining in the cortex and hippocampus of cKO mice was limited to IBA1^+^ microglial cells. The cellular co-expression of BIN1 and IBA1 was assessed by line scan analyses of the two-channel images (Fig. 1B). In the WT, whilst there is a clear overlap of BIN1 and IBA1 signals in a subset of high-intensity peaks signifying microglial BIN1 expression, other BIN1 peaks were devoid of IBA1 signal, indicating BIN1 expression in other cell types (such as oligodendrocytes, which express high levels of BIN1 [4]). In comparison with *Emx*^Cre^-*Bin1* cKO, the high level of parenchymal BIN1 signal was evident in WT mice. Importantly, measurements from the cKO showed a near-perfect alignment of BIN1 and IBA1 signals in high-intensity peaks, demonstrating BIN1 protein expression within the microglia (Fig. 1B). At higher magnification, intense BIN1 immunoreactivity in the perinuclear regions and ramified processes were visible in microglia of *Emx*^Cre^-*Bin1* cKO mice and *Emx*^Cre^ littermates (Fig. 1C). To relate this finding to human microglia, we immunostained post-mortem frontal cortex sections from non-diseased humans in the same manner. BIN1 immunoreactivity was observed in the neuropil and the soma of many cells, some of which were identified as IBA1^+^ microglia. As with mouse brain microglia, BIN1 was localised to perinuclear regions of microglia and the processes in the human brain (Fig. 1D). A line scan across the soma of microglia confirmed the overlap and concomitant intensity changes in the two channels (Fig. 1E). Together, these immunohistochemical studies unequivocally confirm microglial BIN1 expression in both human and mouse brains.

### Characterization of microglial BIN1 isoforms

To identify microglial BIN1 isoforms, we probed immunoblots of lysates from mouse whole-brain homogenates and adult mouse brain-derived CD11b^+^CD45^int^ FACS-purified microglia [41]. In accordance with previous analyses of BIN1 isoforms [5, 11], the blots of mouse brain homogenates revealed predominantly higher molecular weight (~ 75-80 kDa) BIN1 isoforms, representing those containing the CLAP domain (henceforth referred to as BIN1:H), with low-level detection of lower molecular weight (~ 50-55 kDa) isoforms, which lack the CLAP domain (henceforth referred as BIN1:L) (Fig. 1F). In contrast, BIN1 in fluorescence-activated cell sorting (FACS)-isolated microglial lysates corresponded to BIN1:L, with an almost complete lack of the CLAP domain-containing isoforms. Some low-level expression of proteins that migrated between the dominant BIN1:H and BIN1:L isoforms was evident in microglia. These intermediate-sized proteins may represent a post-translational modification of BIN1:L isoforms or possibly low-level alternate splicing of one or more exons that constitute the CLAP domain.

In order to elucidate *Bin1* isoform expression, we performed RT-PCR analysis of FACS-isolated mouse brain-derived microglia and mouse brain. Results from a set of reactions spanning exons 6-10 demonstrated that exon 7 within the BAR domain is excluded in microglial *Bin1* transcripts (Fig. 1G, left). Furthermore, the skeletal muscle-specific exon 11, which codes for a polybasic sequence that confers binding to phosphoinositides and is essential for BIN1-induced membrane tubulation [52], is spliced out from *Bin1* transcripts in adult mouse brain microglia and human induced iPSCs-derived microglia (Fig. S2A). In concurrence with our immunoblot data, RT-PCR across the region spanning exons 12-19 revealed that microglial *Bin1* transcripts predominantly exclude the exons 13-16 corresponding to the CLAP domain, with some level of alternative splicing in this region (Fig. 1G, right). As expected, isoforms containing the CLAP domain and those lacking this domain were amplified in whole-brain RT-PCR (Fig. 1G). We verified our interpretation of *Bin1* splicing in microglia by cloning the microglial RT-PCR products and analysing the amplified regions in individual clones by gel electrophoresis (for predominant splice pattern) and/or sequencing (predominant and non-predominant splice patterns). The vast majority of clones lacked exons 13-17 (Δ CLAP, Δ exon 17), accounting for ~ 90% of *Bin1* transcripts (isoform 10), with low-frequency inclusion of exons 13 and 17 (isoforms 6, 12, and 9; Figs. 1H and S2B).

In order to relate the above findings from murine microglia to human expression, we generated differentiated microglia-like cells (iMG) from human induced pluripotent stem cells (iPSC) [44–46] and extracted protein lysates. Immunoblot analysis demonstrated the presence of BIN1:L isoforms, the lack of BIN1:H isoforms, and the presence of some intermediate-size BIN1-related polypeptides. These cross-species investigations show that the general pattern of BIN1 isoforms expressed in human iPSC-derived microglial cells resembles the BIN1 isoforms found in FACS-isolated adult mouse brain microglia (Fig. 1F).

### BIN1 is a regulator of proinflammatory activation, cytokine production, and neurodegeneration-associated gene expression in primary mouse microglia

**Fig. 2:**
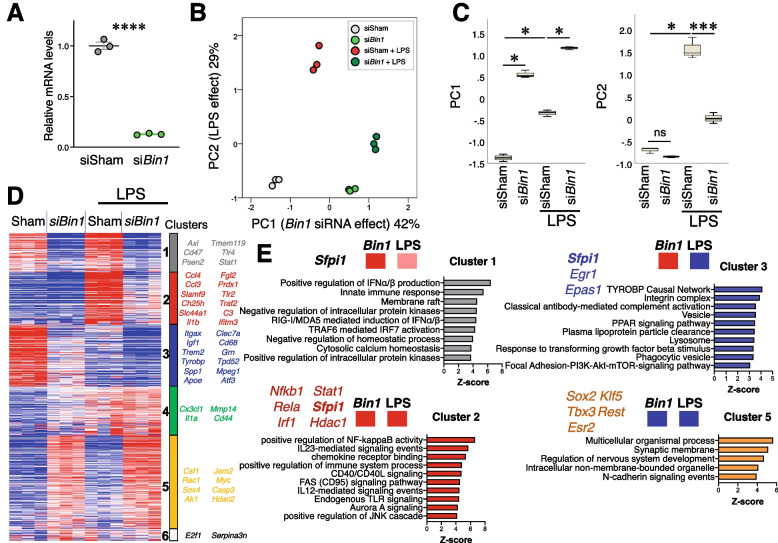
*Bin1* KD in primary microglia dysregulates proinflammatory and PU.1-dependent genes. **A** Bin1 siRNA transfection resulted in > 80% reduction in Bin1 transcripts, as confirmed by qRT-PCR. **B** PCA identified two PCs, which accounted for 71% of the variance in the dataset. PC1 captured the effect of Bin1 loss (42%), while PC3 captured the LPS effect (29%). The LPS effect shown by PC2 was blunted in the absence of Bin1. **C** Both PCs were increased by LPS stimulation. Bin1 KD caused a significant increase in PC1 in resting and LPS-stimulated microglia; Bin1 KD only decreased PC2 during LPS stimulation (* *p* < 0.05, **p < 0.01, ***p < 0.001, Dunn’s). **D** K-means clustering identified six gene clusters, of which five showed distinct patterns of expression based on in vitro manipulations. Cluster 1 was positively regulated by BIN1 in homeostasis, and LPS-stimulated up-regulation was BIN1-dependent. Cluster 2 was positively regulated by BIN1 during LPS stimulation, but its homeostatic regulation was not affected by BIN1. Cluster 3 was positively regulated by BIN1 (during homeostasis and LPS stimulation) but downregulated during LPS stimulation. Cluster 5 was negatively regulated by BIN1 and unaffected by LPS stimulation. Cluster 4 was not regulated by BIN1 but was upregulated during LPS stimulation (not shown in the figure). **E** Gene ontology enrichment analyses (GO, KEGG, Wikipathways included) identified key inflammatory and immune (clusters 1 & 2), homeostatic microglial (cluster 3), and non-microglial-specific (cluster 5) pathways affected by in vitro manipulation of primary microglial cultures. Predicted upstream transcriptional regulators for each cluster are shown, among which Sfpi1 (PU.1) was shared across clusters 1, 2, and 3

Neuroinflammation and microglial activation are common pathological features of several neurodegenerative diseases, including AD. Cultured microglia initiate a robust proinflammatory response when exposed to LPS, an agonist of toll-like receptors, resulting in an altered gene signature and release of proinflammatory cytokines (e.g., IL1b, TNF, and IL6), which are implicated in neurodegenerative diseases [53, 54]. To investigate the potential role of microglial BIN1 in the regulation of homeostatic signaling and inflammatory responses, we manipulated primary microglia in culture. Mouse post-natal microglial cultures were treated with *Bin1* siRNA (or sham siRNA) for 48 h and then with or without LPS (100 ng/ml) for an additional 24 h, and their neuroinflammatory profile was assessed (770-gene NanoString neuroinflammatory gene panel) [31, 48, 55]. LPS stimulation caused a significant upregulation of *Bin1* transcripts (Fig. S3B). *Bin1* siRNA treatment substantially suppressed *Bin1* transcript levels (> 80%, Fig. 2A) without impacting cell viability or morphological response to LPS (Fig. S3A and data not shown). Of 681 genes included in the final analysis (Supplemental Table 1), 513 were differentially expressed at the unadjusted level (ANOVA *p* < 0.05) and 498 genes at the adjusted level (FDR < 5%). The first two principal components (PCs) collectively explained 71% of the variance in the dataset. PC1 predominantly accounted for the *Bin1* knockdown (KD) effect (42% variance), which was largely independent of the LPS effect (Fig. 2B-C). PC2 accounted for the LPS effect (29% variance) and, furthermore, showed that the LPS effect was dampened following the loss of *Bin1* (Fig. 2B-C).

K-means clustering revealed 6 clusters of affected genes, of which 5 showed distinct patterns of regulation by LPS and BIN1 (Fig. 2D). Cluster 1, positively regulated by BIN1 under both resting and LPS-stimulated conditions, was enriched in genes (including *Siglec1*, *C3ar1*, *Fcgr1*, and *Tmem119*) involved in innate immune response, regulation of type I interferon production and signaling, lipid binding, antigen binding, and localization in membrane rafts (Fig. 2E and Fig. S4C). This cluster also contained *Bin1*, confirming the effect of *Bin1* siRNA. Cluster 2 genes were upregulated by LPS and positively regulated by BIN1 and included canonical proinflammatory genes (including *Il1b*, *Marco*, *C3*, and *Irak3*), involved in the regulation of NF-κB signaling, cytokine production, and NLRP3 inflammasome function. Cluster 3 genes were downregulated by LPS and positively regulated by BIN1 and included homeostatic (*Cx3cr1*, *Gpr34*) and DAM genes (*Trem2*, *Tyrobp*, *Spp1*, and *Apoe*) [25] involved in endo-lysosomal function, lipid metabolism, adhesion, and TGFβ signaling (see Fig. S4E). Cluster 4 contained genes upregulated by LPS but independent of BIN1, which are involved in ubiquitin-mediated proteolysis, ribosome and epigenetic regulation, and histone methylation, reflecting the profound effect LPS has on microglia. Cluster 5 genes were generally expressed at lower levels, were negatively regulated by BIN1 independent of LPS stimulation, and included genes involved in synaptic transmission, typically expressed in neurons (see Fig. S4F).

**Fig. 3:**
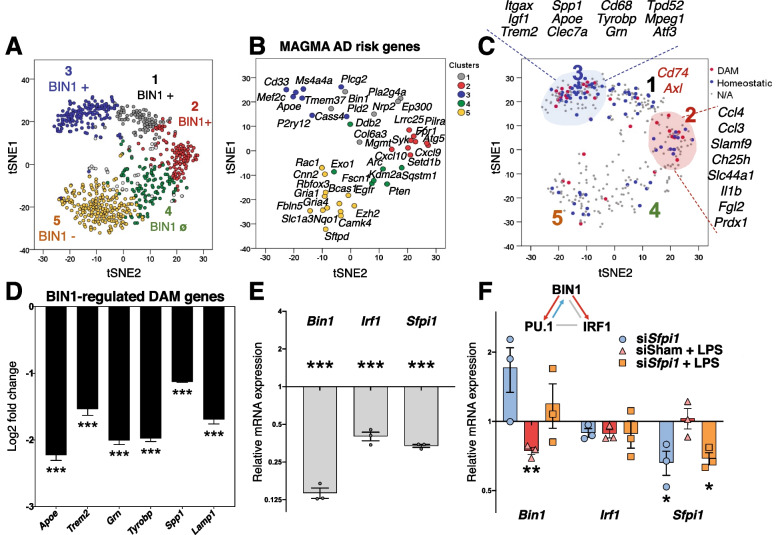
Genes affected by *Bin1* KD in vitro are implicated in AD and regulation of microglial phenotypes. **A** Visualisation of in vitro microglial transcriptomic data using t-SNE shows gene clusters positively (clusters 1-3) or negatively regulated by BIN1 (cluster 5). See the heatmap in Fig. 2D for the cluster color reference. One cluster was unaffected by BIN1 expression (cluster 4). **B** MAGMA of AD-associated risk genes overlapped with our dataset, demonstrating crucial AD-related genes within each cluster that are regulated downstream of microglial BIN1. **C** Critical disease-associated (DAM shown in red) and homeostatic microglial genes (shown in blue) were dysregulated by *Bin1* KD in primary microglia. DAM and homeostatic assignments were based on published literature [25]. **D** qRT-PCR validation confirmed that BIN1 positively regulates several key DAM genes, including *Apoe*, *Trem2,* and *Tyrobp* (i.e., down-regulated by *Bin1* KD) (*p < 0.05, **p < 0.01, ***p < 0.001, two-tailed t-test comparing sham siRNA to *Bin1* siRNA conditions, normalized to *Gapdh*, *n* = 3/condition). **E** *Bin1* KD causes down-regulation of two master transcriptional regulators of microglial phenotypes – *Irf1* and *Sfpi1* (encoding PU.1). **(F)** The plot depicts the q-PCR analysis of the relative change in *Bin1*, *Irf1*, and *Sfpi1* transcript abundance compared to the sham siRNA condition. siRNA KD of *Sfpi1* demonstrates co-dependent regulation between BIN1 and PU.1

We also visualized our gene expression data using T-distributed stochastic neighbor embedding (tSNE) analysis to demonstrate better broad groups of genes positively regulated by BIN1 (overlapped with clusters 1, 2, and 3) and negatively regulated by BIN1 (clusters 4 and 5), identified above by hierarchical clustering analysis (Fig. 3A). In cluster 3 of our dataset, BIN1 positively regulated several DAM genes (Fig. 3C) previously identified by single-cell RNAseq of microglia from mouse models of AD pathology [25]. To ascertain the significance of microglial BIN1-regulated genes in AD pathology, we performed MAGMA of AD-associated genetic risk factors [23]. This analysis revealed substantial overlap with our dataset (Fig. 3B), indicating that several AD risk genes act downstream of BIN1 in microglia. Interestingly, risk genes with high homeostatic expression in microglia (*Apoe*, *Trem2*, *Cd33*, and *Ms4a4a*) were positively regulated by BIN1, whereas neuronal AD risk genes (*Cnn2* and *Gria1*) and autophagy genes (*Sqstm1*) were negatively regulated by BIN1. To validate the regulation of DAM genes by BIN1 in microglia, we performed a qRT-PCR analysis of primary microglia using identical conditions to those used for NanoString studies. We confirmed that BIN1 positively regulated several selected DAM genes, including *Apoe*, *Trem2*, *Grn*, *Tyrobp*, *Spp1,* and *Lamp1* (Fig. 3D and Fig. S3C).

Pathway analysis identified important transcription factors as potential upstream regulators of BIN1-regulated gene clusters (Fig. 2E). PU.1 – a master transcriptional regulator of both microglial development and DAM transition – was predicted upstream of Clusters 1, 2, and 3 genes. Additionally, Cluster 2 genes are known to be regulated by NF-κB, STAT1, IRF1, and HDAC1. Pathway analysis of DAM genes positively regulated by BIN1 indicates ATF3 as an upstream regulator. BIN1 also directly affects *Atf3* transcription in our data, suggesting that ATF3 may serve as an intermediary in BIN1’s control of DAM gene expression. Finally, our in vitro qRT-PCR experiments demonstrate that BIN1 positively regulates transcription of *Sfpi1* (coding PU.1) and *Irf1* (Fig. 3E), two regulators which control numerous microglial genes under homeostatic and inflammatory conditions. Interestingly, we also uncovered a reciprocal relationship between PU.1 and BIN1 (Fig. 3F), adding another layer of complexity to BIN1’s involvement in pathogenic signal dysregulation.

**Fig. 4:**
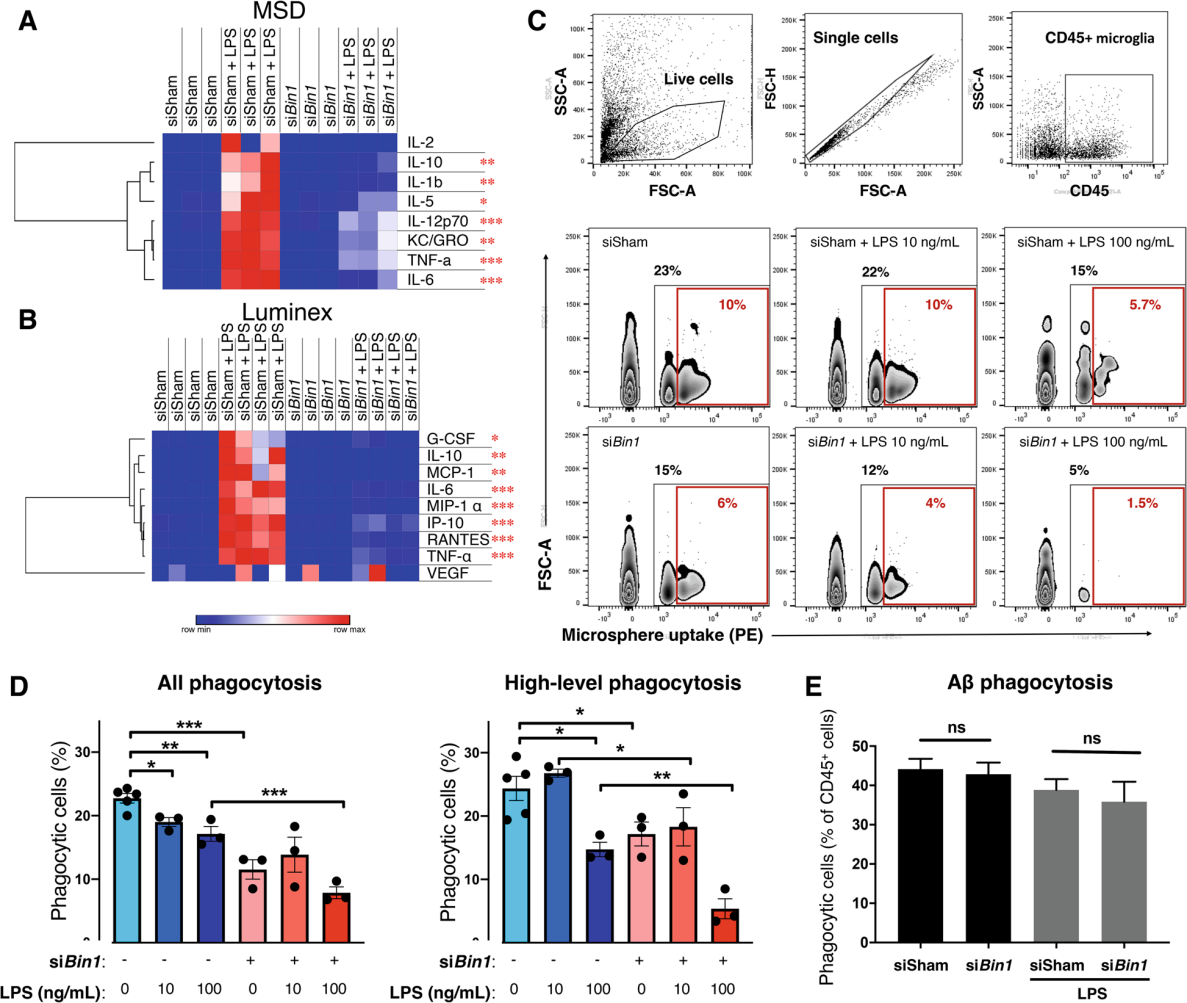
Functional analyses demonstrate BIN1 facilitates inflammation-induced cytokine production, as well as phagocytosis, in primary microglial cultures. **A-B** Bin1 siRNA treatment did not affect cytokine secretion in unchallenged microglial cultures. LPS exposure increased secretion, which was attenuated by the KD of Bin1. **C-D** Flow cytometric analysis of the fluorescent microsphere phagocytosis found that Bin1 reduction impeded the phagocytic capacity of primary microglia, both unchallenged and following LPS stimulation. **E** Phagocytosis of fibrillar Aβ_42_ was unaffected by Bin1 silencing. *, *p* < 0.05; **, *p* < 0.01; ***, *p* < 0.001; by post-hoc t-test with Bonferroni correction for multiple comparisons. Phagocytosis data plotted as mean ± SEM

In light of BIN1’s transcriptional regulation of cytokine gene expression (Fig. 2E, Fig. S4, and Supplemental Tables S1 and S2), we sought to confirm this observation at a functional level. Primary microglia were assayed for cytokine secretion following *Bin1* KD and LPS exposure. We found no differences in cytokine secretion under basal conditions following *Bin1* KD; however, the diminution of *Bin1* expression attenuated LPS-induced increases in the levels of secreted proinflammatory cytokines (i.e., TNF, RANTES, and IL6) across two different assay platforms (MSD and Luminex; Fig. 4A-B). Analysis of transcript levels from the six cytokines included in our NanoString panel demonstrated a similar pattern (Cluster 2) for five of these (*Il1b*, *Tnf*, *Ccl2*, *Ccl3*, *Ccl5*). Of further functional importance, several proteins coded by Cluster 3 genes (*Trem2*, *Tyrobp*, *Cd68*, and *Apoe*; see Fig. 3C) regulate phagocytosis in microglia. To investigate the functional significance of these gene expression changes, we analyzed the phagocytic capacity of primary microglia following *Bin1* KD (Fig. 4C-D). Reduced *Bin1* expression resulted in a decrease in phagocytosis of fluorescent microspheres and augmented the impairment induced by high-dose LPS exposure. However, we observed no effect for *Bin1* loss on the ability of primary microglia to phagocytose fluorescent Aβ_42_ fibrils (Fig. 4E). Overall, our in vitro studies suggest that BIN1 regulates proinflammatory responses, the expression of several neurodegenerative disease-relevant genes, and cytokine production in primary mouse microglia.

### Microglia-specific ablation of *Bin1* mitigates LPS-mediated proinflammatory activation and DAM gene expression profile in vivo

**Fig. 5:**
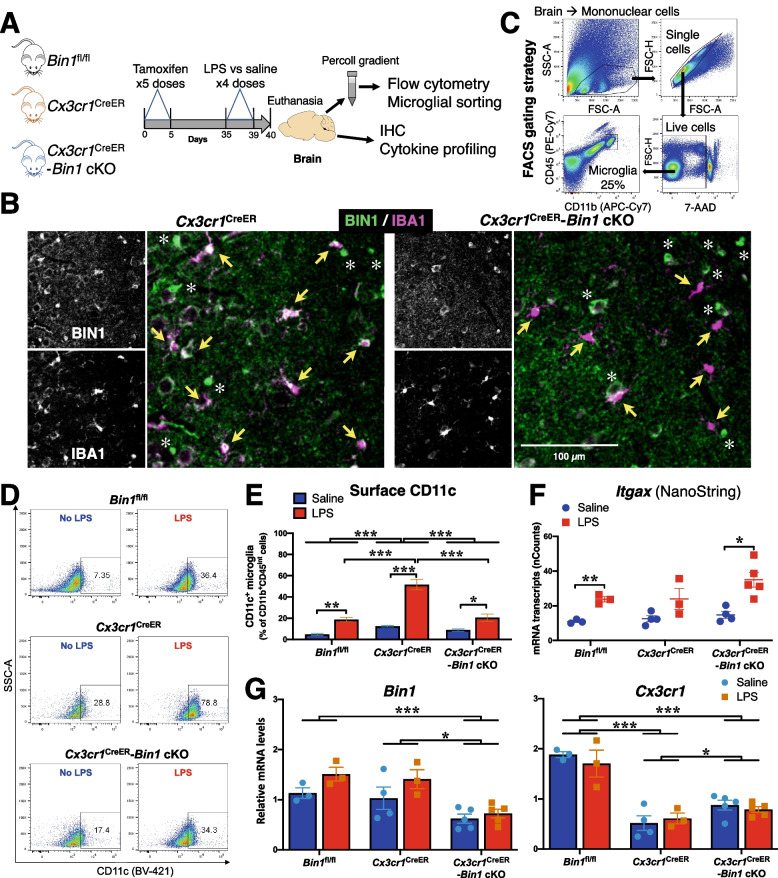
In vivo deletion of *Bin1* affects surface CD11c expression. **A** Experimental strategy for in vivo experiments involved three groups of mice: Bin1^fl/fl^ (WT equivalent), Cx3cr1^CreER^ (primary reference group), and Cx3cr1^CreER^-Bin1 cKO (experimental group). Mice were injected with tamoxifen for 5 consecutive days, then rested for four weeks to allow replenishment of Bin1 expression in peripheral monocytes. Mice then received saline or LPS for four consecutive days, and brains were harvested for flow cytometry / FACS, IHC, and cytokine assays, 24 h after the final injection. **B** Immunofluorescence staining in the piriform cortex demonstrates BIN1 expression in microglia (yellow arrows), oligodendrocytes (asterisks), and synapses (unlabelled) in mice with normal BIN1 expression (Cx3cr1^CreER^). Bin1 was deleted from the microglia of experimental mice (Cx3cr1^CreER^-Bin1 cKO), whilst oligodendrocytes and synaptic BIN1 were unaffected. **C** Mouse brain cells were labelled with APC-Cy7 α-CD11b, PE-Cy7 α-CD45, and BV421 α-CD11c. Single, mononuclear, live cells were gated, and microglia were sorted as CD11b^+^CD45^INT^ population. **D** A representative flow cytometric image of each experimental group is depicted. **E** Flow cytometric analysis demonstrates that LPS administration in vivo caused an increase in the proportion of cells with high surface CD11c expression in all genotypes. The LPS effect was augmented by Cx3cr1 haploinsufficiency (Cx3cr1^CreER^); this additional increase was blunted by microglial Bin1 deletion (Cx3cr1^CreER^-Bin1 cKO). Two-way ANOVA found main effects for genotype (F_2,17_ = 32.98, *p* < 0.001) and LPS (F_1,17_ = 100.9, *p* < 0.001). There was a significant genotype*LPS interaction (F_2,17_ = 16.87, *p* < 0.001). **F** NanoString mRNA counts show that LPS increased Itgax transcript numbers (F_1,16_ = 27.014, *p* < 0.001). No differences between genotypes (F_2,16_ = 3.065, *p* = 0.075) and no genotype*LPS interactions (F_2,16_ = 1.052, *p* = 0.372) were found. Bin1 deletion did not attenuate Itgax transcript numbers. **G** NanoString analysis of mRNA from sorted microglia demonstrates that our cKO system resulted in approximately 50% decrease in microglial Bin1 expression (F_2,17_ = 13.14, *p* < 0.001), which was not affected by LPS (F_2,17_ = 0.712, *p* = 0.505), despite the main effect for LPS increasing Bin1 transcripts (F_1,17_ = 5.853, *p* = 0.027). Analysis of Cx3cr1 transcript numbers found a main effect for genotype (F_2,17_ = 43.802, *p* < 0.001), with post-hoc differences between Bin1^fl/fl^ with Cx3cr1^CreER^ (*p* < 0.001), Bin1^fl/fl^ with Cx3cr1^CreER^-Bin1 cKO (*p* < 0.001), and Cx3cr1^CreER^ with Cx3cr1^CreER^-Bin1 cKO (*p* = 0.043) demonstrating that the reduction in Cx3cr1 expression in the Cre line was partially attenuated by Bin1 deletion. No main effect for LPS treatment (F_1,17_ = 0.303, *p* = 0.589) and no genotype*LPS interaction (F_2,17_ = 0.515, *p* = 0.606) were found. All by two-way ANOVA. *, *p* < 0.05; **, *p* < 0.01; ***, *p* < 0.001; by post-hoc t-test with Bonferroni correction for multiple comparisons. All data plotted as mean *±* SEM

Considering the differences in microglial phenotypes between culture conditions and in vivo, we proceeded to investigate the effects of microglia-specific *Bin1* deletion on the mouse brain microglial transcriptome under homeostatic and inflammatory (systemic LPS) conditions. We employed an inducible conditional *Bin1* knockout strategy by crossing *Cx3cr1*^*tm2.1(cre/ERT2)Litt*^/WganJ [56] (heterozygous animals of this line are referred to as *Cx3cr1*^CreER^) with *Bin1*^fl/fl^ mice. Experimental groups included *Bin1*^fl/fl^ as the wild-type equivalent, *Cx3cr1*^CreER^ as the primary reference group (as these mice only have one functional *Cx3cr1* allele), and *Cx3cr1*^CreER^;*Bin1*^fl/fl^ (*Cx3cr1*^CreER^-*Bin1* cKO) as the experimental group. Following 5 days of daily tamoxifen injections, mice were rested for a four-week interval to allow replenishment of peripheral monocytes/macrophages (which also express *Cx3cr1*). Following this, LPS was administered for four consecutive days to induce a well-characterised proinflammatory microglial response [57, 58], and mice were euthanized 24 h after the final injection (Fig. 5A). As expected, LPS induced a sickness response of hypothermia and weight loss. Interestingly, we observed a trend towards dampened hypothermic responses in *Bin1* cKO mice without affecting weight loss (Fig. S5B-E).

LPS administration did not cause a significant change in *Bin1* expression in FACS-isolated brain microglia as quantified by NanoString analysis (Fig. S5G), and no change in *Bin1* splicing was detected by RT-PCR (Figs. 1G, S2A, and S5H). Using immunofluorescence staining, we confirmed BIN1 expression in IBA1^+^ microglia of *Cx3cr1*^CreER^ but not *Cx3cr1*^CreER^-*Bin1* cKO mice (Fig. 5B). Whilst LPS administration induced a morphological transition into an amoeboid phenotype in WT (*Bin1*^fl/fl^) microglia, *Cx3cr1* haploinsufficient microglia presented a hyper-ramified morphology in response to LPS (Fig. S6A). Strikingly, *Cx3cr1*^CreER^-*Bin1* cKO microglia appeared to fully retain resting morphology (Fig. S6A), suggesting a functional inability to respond to inflammatory conditions. However, key parameters of microglial morphology were not significantly affected, demonstrating the highly variable morphological response of these heterogeneous cells, with significant differences between brain regions (Fig. S6B-C). LPS administration did not seem to affect BIN1 expression or localization in microglia, as detected by immunofluorescence histology staining of *Bin1*^fl/fl^ mouse brains (Fig. S5F).

Flow cytometry was performed on mononuclear cells isolated from brains to measure the surface expression of CD11b, CD45, CD11c, and Ly6c. CD11b^+^CD45^int^ microglia were FACS-purified and processed for NanoString transcriptomic profiling (Fig. 5A and C). Based on Iba1 immunofluorescence staining and flow cytometry studies, we observed no difference in microglial density or numbers across the genotypes (Fig. S6A and data not shown). Amongst CD11b^+^CD45^int^ microglia, there was a significant increase in the proportion of CD11c^+^ microglia following LPS treatment, which was most apparent in *Cx3cr1*^CreER^ mice (Fig. 5D and E), likely attributable to *Cx3cr1* haploinsufficiency. In contrast to *Cx3cr1*^CreER^ mice, the additional loss of *Bin1* (*Cx3cr1*^CreER^-*Bin1* cKO) abrogated this LPS effect (Fig. 5D and E). Thus, high-level surface CD11c expression, a feature of microglial activation and signature of the DAM phenotype [21, 25, 59] (several genes of which are upregulated following LPS exposure [60]), becomes apparent in microglia in the LPS-induced neuroinflammation model and appears to be moderated by CX3CR1 signaling. Collectively, these findings indicate that BIN1 positively regulates cell surface CD11c levels in microglia and may control the induction of the DAM phenotype following systemic LPS administration.

**Fig. 6:**
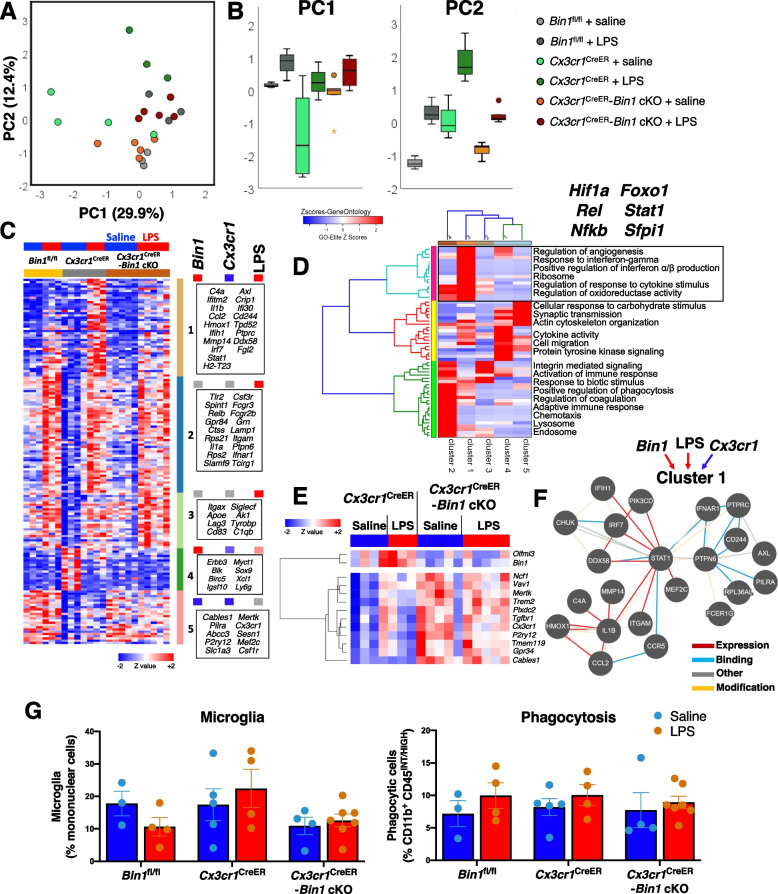
In vivo microglia-specific loss of *Bin1* dampens the proinflammatory microglial response. **A** PCA of gene expression data from FACS-purified mouse brain microglia from in vivo *Bin1* cKO studies identified two PCs which accounted for 42% of the variance in the data. **B** PC1 (effect of LPS regardless of genotype) explained 29.9% of the variance, whilst PC2 (LPS effect impacted by genotype) explained 12.4% of the variance and exemplified the pattern of *Bin1* cKO mitigating dysregulation by Cx3cr1 haploinsufficiency. **C** K-means clustering identified five clusters of genes affected in our dataset. Cluster 1 genes were upregulated during LPS stimulation, dependant on BIN1. Cluster 2 was upregulated by LPS stimulation and positively regulated by BIN1 (downregulated by *Bin1* cKO). Cluster 3 was upregulated by LPS independent of BIN1. Cluster 4 was downregulated by LPS and positively regulated by BIN1 in unstimulated conditions. Cluster 5 genes were negatively regulated by BIN1, counter to CX3CR1. **D** Gene ontology enrichment analysis identified interferon-response pathways regulated by cluster 1 genes. **E** Thirteen microglial genes were suppressed by BIN1 (upregulated by *Bin1* cKO) independent of LPS inflammation, including homeostatic genes *P2ry12*, *Tmem119,* and *Tgfbr1*. **F** Pathway analysis suggests STAT1 signaling may regulate expression of cluster 1 genes (nature of the interaction between genes is shown based on color scheme shown in the key.) **G** Analysis of microglia numbers found no main effects for genotype (F_2,21_ = 2.614, *p* = 0.097), LPS treatment (F_1,21_ = 0.002, *p* = 0.966), or no genotype*LPS interaction (F_2,21_ = 1.192, *p* = 0.323) (by two-way ANOVA). Phagocytic capacity was not affected by LPS (F_1,21_ = 1.939, *p* = 0.178) or genotype (F_2,21_ = 0.121, *p* = 0.887) in *Bin1* cKO studies, and no genotype*LPS interactions was found (F_2,21_ = 0.101, *p* = 0.904) (by two-way ANOVA). Data plotted as mean *±* SEM. For associated physiological data and immunohistochemistry data, see Fig. S5B-F

Inflammatory gene expression data from FACS-purified microglia were then analyzed for 511 transcripts (Supplemental Table S3). Microglia isolated from *Cx3cr1*^CreER^-*Bin1* cKO animals had approximately 50% lower *Bin1* levels than *Cx3cr1*^CreER^ controls (Fig. 5G). The efficiency of *Bin1* loss did not vary between sexes or by LPS-treatment (data not shown and Fig. 5G). As expected, microglia from *Cx3cr1*^CreER^ mice showed lower *Cx3cr1* expression, demonstrating the *Cx3cr1* haploinsufficiency of this mouse line (Fig. 5G). PCA showed that two PCs explained 43% of the variance in the data (PC1 29.9%, PC2 12.4%) (Fig. 6A). PC1 captured the LPS effect that was relatively similar across all three genotypes. PC2 captured LPS responses that were modified by *Cx3cr1* genotype as well as by *Bin1* deletion. At baseline, *Cx3cr1*^CreER^ mice showed higher levels of activation when compared with WT (*Bin1*^fl/fl^) mice. The LPS response captured by PC2 was most pronounced in *Cx3cr1*^CreER^ mice, consistent with the previous characterization that signaling through CX3CR1 controls microglial activation [61]. Interestingly, the loss of *Bin1* mitigated the heightened LPS response elicited by *Cx3cr1* haploinsufficiency. These high-level transcriptomic findings align with our flow cytometric results suggesting that, during inflammation, the CD11c^+^ DAM phenotype is facilitated by BIN1.

Of 511 genes included in the analysis, 164 genes show group-wise differential expression (ANOVA *p* < 0.05, Supplemental Table S3). K-means clustering of these differentially expressed genes identified 5 clusters with distinct patterns of expression (Fig. 6C). Cluster 1, which contains proinflammatory genes (including *C4a*, *Il1b*, *Ccl2*, *Irf7*, and *Stat1*), was upregulated following LPS, while the loss of *Bin1* suppressed this response. Therefore, cluster 1 genes were positively regulated by BIN1 specifically under proinflammatory conditions, corroborating the aforementioned in vitro phenotype from *Bin1* siRNA KD. Cluster 2 genes demonstrated a uniform LPS effect across all genotypes and included some canonical proinflammatory genes known to be upregulated by LPS (e.g., *Tlr2*, *Il1a*, and *Fcgr3*), as well as *Apoe*, the ε4 allele variant of which is the highest predictor of LOAD risk [62]. Cluster 3 genes showed a pattern similar to Cluster 2 without an apparent BIN1 dependence, and included *Itgax* (which encodes CD11c), *Tyrobp*, and *Rpl9*. While increased *Itgax* expression by LPS was consistent with flow cytometric findings of an increase of CD11c^+^ microglia in LPS-treated mice (Fig. 5D and E), the unabating expression of *Itgax* following *Bin1* deletion despite a decrease of CD11c^+^ microglia was surprising (Fig. 5F). This finding suggests that post-transcriptional or post-translational control of CD11c expression, or surface localization, requires BIN1 function. Cluster 4 genes were suppressed by LPS and showed amelioration with the additional *Bin1* deletion, indicative of positive regulation by BIN1 during inflammation. Cluster 5 contains genes that were negatively regulated by both BIN1 and CX3CR1, irrespective of LPS treatment. A summary of overall trajectories of changes in gene expression at the cluster level is presented in Fig. S7A-B. Additionally, we identified 13 genes (including *Olfml3*, *Tmem119*, *Mertk*, *Trem2,* and *P2ry12*) regulated by BIN1 independent of the state (Fig. 6E).

Gene set enrichment analyses of Cluster 1 genes suggested positive regulation of type I interferon (α/β) expression and production (Fig. 6D). In addition, the BIN1-regulated Cluster 1 was enriched in ribosomal genes (*Rpl28*, *Rpl29*, *Rps10*, and *Rps9*), cytokines, and response to IFNγ genes (*Il1b*, *Ccl2*, *Ccl5*, and *Stat1*), as well as regulation of oxidoreductase activity (*Apoe*, *Il1b*, and *Slamf8*). Thirty-three of the LPS-upregulated genes were suppressed at least 1.5-fold following *Bin1* deletion. An analysis of the known interactions between these genes and their encoded proteins is shown in Fig. 6F.

Overall, our in vivo studies suggest that BIN1 somewhat counteracts *Cx3cr1* haploinsufficiency. The loss of CX3CR1 signaling has previously been shown to increase phagocytosis of fluorescent microspheres [63]. We, therefore, sought to relate the loss of *Bin1* in microglia with microglial functionality, focusing on their ability to phagocytose fluorescent microspheres [50] and asked whether the additional loss of *Bin1* was sufficient to reverse the effect of *Cx3cr1* haploinsufficiency. However, we found that neither *Cx3cr1* haploinsufficiency nor deletion of microglial *Bin1* had any impact on phagocytosis under the assay conditions employed in this study, and LPS also failed to impact this cellular function (Fig. 6G). The lack of any observed effect of FACS-sorted microglia lacking *Bin1* on phagocytosis was somewhat consistent with the modest effects observed in cultured microglia following *Bin1* siRNA KD.

We also measured inflammatory cytokine levels in brain homogenates (sampled from the frontal cortex) from all experimental mice by Luminex (32 cytokine panel) (Fig. S7C). LPS increased eotaxin, MIG, and IP-10 levels in all genotypes with no effect of *Bin1* loss. While the effect of *Bin1* deletion within unstimulated and LPS-stimulated groups was not statistically significant, we observed global effects of *Bin1* deletion, independent of LPS treatment. As compared to *Cx3cr1*^CreER^ mice, *Cx3cr1*^CreER^-*Bin1* cKO mice had higher levels of IFNγ (*p* = 0.017), IL4 (*p* = 0.026) and IL7 (*p* = 0.014).

### BIN1 may mediate its transcriptomic effects in microglia by impacting type 1 interferon signaling

**Fig. 7:**
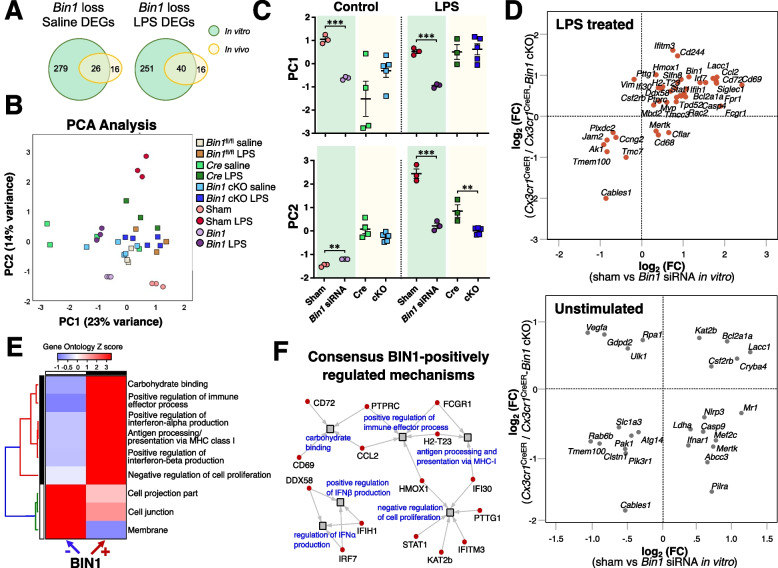
Concordance analysis between in vitro and in vivo NanoString datasets reveals a common pattern of microglial gene regulation by BIN1. **A** The Venn diagrams illustrate the overlapping DEGs in the in vitro and in vivo datasets under basal and LPS-stimulated conditions. **B** PCA demonstrates two principal components account for 37% of the variance in the combined dataset. **C** The PCA results show the similarities and differences between the in vitro and in vivo systems. **D** Whereas low concordance between in vitro and in vivo datasets was visualised from unstimulated microglia, LPS-stimulated cells showed higher concordance in gene expression between our model systems. **E-F** Gene ontology analysis of genes concordantly regulated in the in vitro and in vivo datasets found interferon- and membrane-related pathways to be regulated by BIN1

Our in vitro studies showed robust effects of *Bin1* loss on microglial gene expression and inflammatory cytokine production. However, post-natal microglia do not entirely recapitulate adult microglial responses [64]. While our in vivo experiments overcome these limitations of in vitro studies, the effect of *Cx3cr1* haploinsufficiency complicates interpretations from in vivo microglia-selective *Bin1* deletion. Despite these limitations, we observed notable overlap in gene ontologies and patterns of BIN1-mediated gene regulation from in vitro and in vivo studies. In order to identify the most robust and concordant findings emerging from our in vitro and in vivo data, we performed a combined analysis of NanoString datasets derived from both sets of experiments. A set of 498 genes with expression above threshold across both datasets were included in this analysis. The Venn diagrams show the number of shared DEGs under basal and LPS-stimulated conditions in the in vitro and in vivo datasets (Fig. 7A). PCA of these shared gene sets revealed suppression of LPS-induced transcriptomic changes in microglia following LPS stimulation as the common feature following the reduction of *Bin1* expression (Figs. 7B and C). Under non-stimulated conditions (sham siRNA vs. *Bin1* siRNA in vitro, and *Cx3cr1*^CreER^ vs. *Cx3cr1*^CreER^-*Bin1* cKO microglia from in vivo studies, all without LPS treatment), we observed few concordant genes regulated by *Bin1* in both datasets (Fig. 7D). However, under LPS stimulated conditions (sham siRNA+LPS vs. *Bin1* siRNA+LPS in vitro, and *Cx3cr1*^CreER^ + LPS vs. *Cx3cr1*^CreER^-*Bin1* cKO + LPS microglia from in vivo studies), we observed greater concordance between the two model systems (Fig. 7D). We combined these concordant lists of BIN1-regulated genes (31 genes positively regulated including *Cd69*, *Ccl2*, *Irf7*, and *Ifitm3*; and 13 genes negatively regulated including *Cables1* and *Tmem100*) and performed GO enrichment analysis to identify BIN1-regulated ontologies in microglia. BIN1 was found to be a positive regulator of carbohydrate binding, type I interferon and immune pathways, antigen presentation via MHC-I, and a negative regulator of cell proliferation. BIN1 also negatively regulated genes located at cell projection, cell junction, and membrane (Fig. 7E). Specific BIN1-regulated genes involved in type I interferon signaling included *Ddx58*, *Ifih1*, *Irf7*, *Ifi30,* and *Ifitm3* (Fig. 7F).

### Inflammatory upregulation of *Ifitm3* is dependent on BIN1 expression

**Fig. 8:**
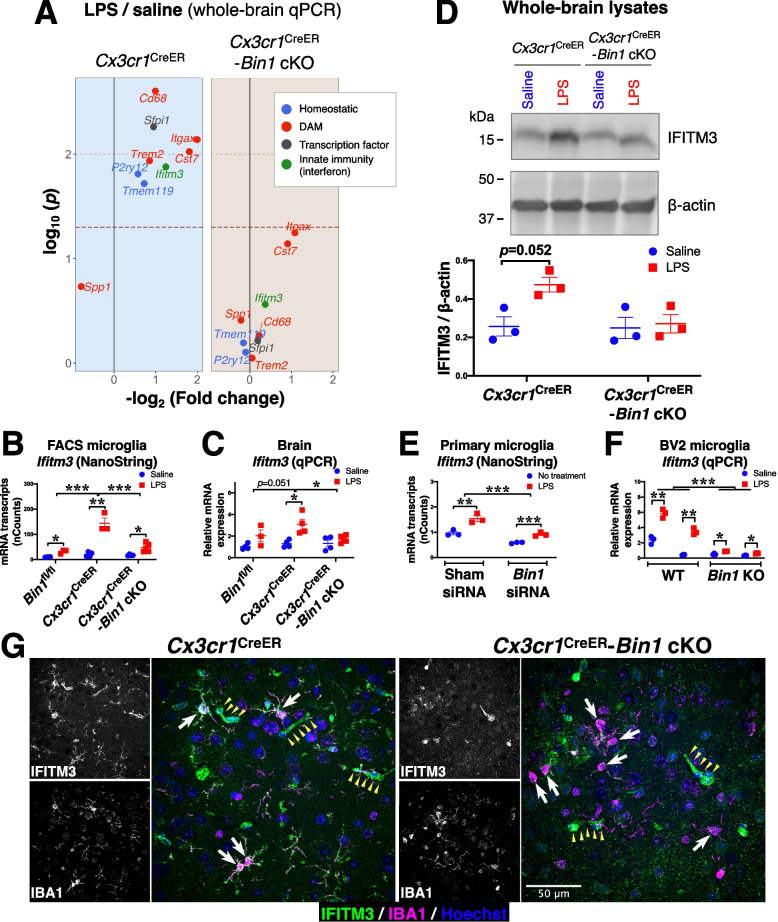
LPS-induced up-regulation of IFITM3 in microglia is dependent on BIN1. **A** qRT-PCR analysis of whole-brain cDNA found inflammation-induced upregulation of key homeostatic and DAM genes are dependent on BIN1. Upregulation of a crucial myeloid transcription factor (Sfpi1), as well as an interferon-induced innate immune gene (Ifitm3), were also BIN1-dependent. Raw dataset is provided in Fig. S8. **B** NanoString analysis of transcripts in FACS-isolated microglia demonstrates an up-regulation of Ifitm3 following in vivo LPS injections. This effect is augmented in Cx3cr1^CreER^ microglia and is dependent on BIN1. Main effects were found for genotype (F_2,16_ = 26.538, *p* < 0.001) and LPS treatment (F_1,16_ = 66.105, *p* < 0.001), and a significant genotype*LPS interaction was found (F_2,16_ = 20.609, *p* < 0.001) (by two-way ANOVA). Post-hoc pairwise comparisons found Cx3cr1^CreER^ to be different from both Bin1^fl/fl^ (*p* < 0.001) and Cx3cr1^CreER^-Bin1 cKO (*p* < 0.001) (with Fisher’s LSD applied). **C** qRT-PCR analysis of whole-brain transcripts validated the pattern of microglial expression. A main effect for LPS treatment was found (F_1,18_ = 17.497, *p* < 0.001), but the effect for genotype (F_2,18_ = 3.189, *p* = 0.065) and the genotype*LPS interaction (F_2,18_ = 2.734, *p* = 0.092) failed to reach significance (by two-way ANOVA). Despite this, post-hoc pairwise comparisons found Cx3cr1^CreER^ to be different from Cx3cr1^CreER^-Bin1 cKO (*p* = 0.036). However, the comparison with Bin1^fl/fl^ genotype failed to reach significance (*p* = 0.051) (with Fisher’s LSD applied). **D** Immunoblot analysis of whole-brain lysates confirmed the transcriptional regulation results in similar IFITM3 protein level changes. Whilst a main effect for LPS treatment was found (F_1,8_ = 6.156, *p* = 0.038), genotype (F_1,8_ = 4.788, *p* = 0.06) and the genotype*LPS interaction (F_1,8_ = 4.126, *p* = 0.077) failed to reach significance in our data (by two-way ANOVA). **E** NanoString analysis of transcripts in primary cultured microglia shows Ifitm3 expression is blunted in Bin1 KD cells. Main effects for siRNA treatment (_F1,8_ = 53.326, *p* < 0.001) and LPS (F_1,8_ = 43.226, *p* < 0.001) were found. There was no siRNA*LPS interaction (F_1,8_ = 3.137, *p* = 0.115). **F** CRISPR-edited BIN1 BV2 KO microglia validate that Ifitm3 upregulation in response to LPS stimulation is impaired in Bin1 KO cells, with main effects for Bin1 (F_1,20_ = 44.503, *p* < 0.001) and LPS (F_1,20_ = 23.945, *p* < 0.001), and a significant Bin1*LPS interaction (F_1,20_ = 16.023, *p* < 0.001). **G** Immunofluorescence detection of IFITM3 in mouse brain demonstrates that IFITM3 expression throughout the LPS-treated Cx3cr1^CreER^ microglia but not in Cx3cr1^CreER^-Bin1 cKO. Note that microglia are indicated by white arrows, and IFITM3 labelling of blood vessels is indicated by small yellow arrowheads. *, *p* < 0.05; **, *p* < 0.01; ***, *p* < 0.001; by post-hoc t-test with Bonferroni correction for multiple comparisons. All data plotted as mean ± SEM

In light of the profound effect of *Bin1* deletion on gene transcription, we validated key homeostatic and DAM genes identified in our NanoString panel – as well as *Cst7*, a DAM gene – by qRT-PCR (Fig. 8A and Fig. S8). We also found that inflammatory upregulation of transcription factor *Sfpi1* (identified by network analysis) depended on BIN1 (Fig. 8A and Fig. S8). Based on its localization in the cytosol and processes, we reasoned that microglial BIN1 likely functions as a cytosolic adaptor protein that regulates receptor dynamics in the context of endocytosis and recycling, rather than a transcriptional regulator. We, therefore, sought to determine whether BIN1 regulates signaling via type 1 interferon pathways. One key gene uncovered in our datasets is the lysosome-associated restriction factor *InterFeron Induced TransMembrane protein 3* (*Ifitm3*), whose expression is activated by type I and type II interferon signaling. To validate our NanoString data from FACS-isolated microglia (Fig. 8A-B), we performed a qRT-PCR analysis of whole-brain RNA isolated from *Cx3cr1*^CreER^ and *Cx3cr1*^CreER^-*Bin1* cKO mice following LPS challenge (or saline injection). We found an LPS-induced upregulation of *Ifitm3* transcripts in *Cx3cr1*^CreER^ mice, which was significantly attenuated by *Bin1* deletion (Fig. 8A-C). We further validated this finding at the protein level in immunoblot analysis of whole-brain protein lysates (Fig. 8D). Transcript levels from cultured microglia quantified by NanoString analysis confirmed that the *Ifitm3* upregulation is blunted by *Bin1* knockdown in the absence of *Cx3cr1* haploinsufficiency (Fig. 8E). In order to further validate this effect without the limitations of *Cx3cr1* haploinsufficiency (in vivo) or incomplete transient *Bin1* reduction (siRNA KD), we generated stable pools of *Bin1* KO microglial cells by CRISPR/Cas9-mediated gene editing in the BV2 cell line (Fig. S9). *Bin1* KO cells displayed a remarkably blunted *Ifitm3* response to LPS treatment (Fig. 8F). Strikingly, immunofluorescence labelling of mouse brain sections with an IFITM3 antibody demonstrated intense IFITM3 immunoreactivity throughout the microglial soma and ramifications of *Cx3cr1*^CreER^ mice following LPS challenge, with little, if any, LPS-mediated upregulation in the absence of BIN1 expression (Fig. 8G).

Collectively, our congruent findings derived from transcriptomic profiling of microglia and experimental validation studies show that microglial BIN1 positively regulates key elements of DAM phenotype transformation and type 1 interferon networks, which are likely to be independent of in vitro versus in vivo differences and independent of *Cx3cr1* haploinsufficiency.

## Discussion

Our study demonstrates, for the first time, that BIN1 is a key regulator of microglial gene expression and functionality, both in homeostatic and inflammatory conditions. By transcriptional profiling of cultured microglia challenged with LPS, we identify BIN1 as a regulator of proinflammatory activation, cytokine production, and neurodegeneration-associated gene expression. The in vitro results were confirmed by analysing FACS-isolated adult brain microglia from WT and cKO mice and showing that microglial BIN1 is a key regulator of LPS-mediated proinflammatory activation and DAM gene expression in vivo.

High-level BIN1 expression in microglial transcriptomics and proteomics datasets has been reported previously [16–18]. Still, there have been only two reports of cellular co-localization of BIN1 with microglial markers. One identified IBA1-positive cells expressing BIN1 in immunohistochemical analysis of post-mortem brain tissue from patients with AD [8]. Another study reported detection of BIN1 isoforms 12 and 6 in the nucleus of CD45^+^ microglia using antibodies raised against BIN1 exons 11 and 13; notably, the major microglial BIN1 isoform 10 lacking both exons 11 and 13 was not observed by immunostaining in the previous study [65]. In this regard, it is worth mentioning that exon 11, which codes for a polybasic sequence that confers binding to phosphoinositides and is essential for BIN1-induced membrane tubulation [66], is spliced out from *Bin1* transcripts in adult mouse brain microglia and human induced iPSCs-derived microglia (Fig. S2A). By generating mice lacking BIN1 expression in excitatory neurons and oligodendrocytes, we have unequivocally demonstrated BIN1 expression in microglia. The identification of BIN1 isoform 10 in our study as the most abundant *Bin1* transcript in mouse brain microglia is consistent with the human brain microglia RNAseq data imputed to BIN1 isoforms [65]. However, our demonstration of BIN1 localization in the perinuclear region and microglial processes is at odds with the earlier report of BIN1 in microglial nuclei [65]. The analyses of BIN1 in *Emx*^Cre^-*Bin1* cKO mice by immunostaining (this study) and immunoblots [5] indicate that microglial BIN1 accounts for only a minor fraction of all BIN1 expressed in the brain. Nevertheless, it is worth noting that a large-scale cell-type-specific promoter–enhancer interaction study identified a microglia-specific enhancer in the human *BIN1* gene, which contains the AD-risk variant rs6733839 [67]. A more recent study predicted the risk variant rs6733839 to facilitate the binding of the transcription factor MEF2C to the *BIN1* enhancer in microglia, increasing *BIN1* expression [68]. Thus, the AD association signal at BIN1 appears to be microglial cell-type-specific.

Specific BIN1 isoforms might participate in divergent functions in relation to membrane dynamics, including endocytosis. Neuronal BIN1 regulates synaptic vesicle release [5] and limits the inter-neuronal tau spread in cultured neurons by regulating endocytic uptake of pathogenic tau [10]. Compared to neuronal BIN1 isoforms, mouse brain microglial BIN1 and human iPSC-derived microglial BIN1 isoforms lack the central CLAP domain, a region conserved between BIN1 and its homolog Amphiphysin 1, which contains the sites for clathrin and AP2 adaptor binding [69]. The CLAP domain is important for BIN1’s function in clathrin-mediated endocytosis; however, all microglial BIN1 isoforms have an SH3 domain, which can interact with the proline-rich domain of dynamin I [70]. Thus, a role for microglial BIN1 in endocytosis could not be excluded solely based on the lack of the CLAP domain. In non-neuronal cells, the depletion of BIN1 does not impede transferrin uptake by endocytosis but rather delays endocytic recycling [71, 72]. Our finding that the loss of BIN1 expression has little effect on microglial phagocytosis shows congruence with a previous study that assessed phagocytosis in *Bin1* KO macrophages and found that BIN1 did not have a functional role in endocytosis or phagocytosis in these immunoregulatory cells [71]. The inclusion of exon 13 (referred to as exon 12A in earlier publications) and exon 17 sequences in a subset of microglial *Bin1* transcripts (albeit at low frequency) may have implications for cell cycle regulation. Indeed, it has previously been reported that the inclusion of exon 13 abrogates BIN1’s binding to the transcription factor E2F1, inhibiting cell cycle progression [73, 74]. Furthermore, exon 13 contains a class I SH3-binding motif (PxxP), which can engage in an intramolecular interaction with BIN1’s own SH3 domain, thereby sequestering its interactions with cMYC [75], as well as potentially impeding interactions with other SH3 domain-dependent partners (e.g., dynamin and tau). Additionally, exon 17 encodes half of the c-MYC binding motif of BIN1, which regulates c-MYC-mediated transformation and apoptosis [76]. The microglia-specific functions of individual BIN1 isoforms, including the minor isoforms containing exons 13 and 17, presents an exciting area for future functional investigations.

Results of our neuroinflammatory transcriptional profiling indicate that BIN1 is a homeostatic microglial regulator that has a non-redundant role in the activation of immune responses upstream of the transcription factor PU.1, which is crucial for microglial viability [77]. PU.1 is a master regulator of microglial gene expression and transition to DAM phenotypes [32, 78–81]. Moreover, PU.1 regulates the expression of several AD-related microglial genes [32], and lower PU.1 expression in myeloid cells has been reported to delay AD onset [82]. Importantly, we have shown that BIN1 regulates *Sfpi1*, which codes PU.1. Interestingly, our consensus in vitro and in vivo finding that BIN1 did not affect microglia numbers suggests that BIN1 function might not be essential for microglial maintenance and survival. Overall, our finding that BIN1 positively regulates PU.1 transcription may suggest that BIN1 expression could be a significant determinant of microglial phenotype and pathology outcomes, particularly in AD, a hypothesis that warrants future investigations.

A limitation of our in vivo model system relates to the inherent *Cx3cr1* haploinsufficiency in *Cx3cr1*^CreER^ mice which by itself is known to impact microglia via premature upregulation of an aging transcriptomic phenotype, inclusive of some disease-associated microglial genes observed in AD pathology [83]. This is exemplified by the differences between *Cx3cr1*^CreER^ mice and the WT equivalent (*Bin1*^fl/fl^) group. This effect of *Cx3cr1* haploinsufficiency may partly explain some lack of concordance between in vitro and in vivo effects of *Bin1* loss in microglia observed in our studies. Differences in responses between primary post-natal microglia in culture versus adult microglia in their native state in the brain, as well as direct effects of LPS on microglia in vitro, and indirect effects of LPS on microglia in vivo, may have also contributed to differences between the in vitro and in vivo experimental paradigms. Despite these differences, we observed several LPS-induced inflammatory genes and pathways that were BIN1-dependent in both model systems (Fig. 7A and D). This consensus analysis of our in vitro and in vivo transcriptomic findings identified a novel role for BIN1 in regulating the type 1 interferon response in microglia. We found that manipulating BIN1 in microglia impacted the expression of interferon regulatory factors (IRFs). IRFs are a family of transcriptional regulatory proteins that translocate to the nucleus in response to signaling events triggered by pathogen recognition receptors. Nuclear-translocated IRFs then regulate the expression of distinct groups of genes involved in immune responses and immune cell development. For example, IRF1, IRF5, and IRF8 play essential roles in proinflammatory responses [84–88], whereas IRF2 and IRF4 regulate anti-inflammatory responses [89]. IRF8 is a critical regulator of microglial motility [90], and IRF7 and IRF8 regulate microglial homeostasis and reactivity [87, 91]. IRF7 is a master signaling regulator of the interferon-dependent immune response [92]. The interferon signaling pathway is activated concomitantly with neuroinflammation in multiple mouse models of AD amyloid pathology [93]. Moreover, IRF7 expression in the human brain is highly correlated with AD clinical dementia, Braak score, and neuritic amyloid burden [93]. In our in vitro studies, we found that loss of BIN1 in microglia decreased expression of IRF1 and IRF7 genes without affecting IRF, IRF3, IRF4, and IRF8 expression. In vivo, loss of microglial BIN1 dampened the upregulation of IRF7 by systemic LPS, mirroring in vitro findings, providing a link between BIN1 function and type 1 interferon signaling in microglia.

Our results further show that *Ifitm3* – one of the genes stimulated by type I interferons, as well as by proinflammatory cytokines that are critical mediators of the host innate immune response, including IL-1b, IL-6, and TNF [35] – is positively regulated by BIN1, both in vitro and in vivo. A recent publication found IFITM3 gene networks to be enriched in tau tangle-containing neurons and peripheral mononuclear cells from AD patients [38]. Expression of IFITM3 is also upregulated in microglia of a transgenic model of AD amyloid pathogenesis [33, 94]. In addition, IFITM3 is upregulated in a transient ‘interferon responsive’ microglial subset that expands during cortical remapping in a partial whisker lesion model. In this microglial population, IFITM3 localised to early phagosomes and was found necessary for phagosome maturation [94]. Interestingly, IFITM3 is necessary for lysosome acidification in macrophages [34, 35]. Further, lysosomes in homeostatic microglia are weakly acidic (~pH 6) compared to peripheral macrophages (~pH 5), and inflammatory activation causes acidification of microglial lysosomes, allowing them to degrade fibrillar Aβ [95]. Thus, an investigation into the functional role of IFITM3 in microglial lysosomes is imperative to elucidate pathology-specific cellular functions regulated by BIN1.

Of crucial importance, several genes transcriptionally regulated by BIN1 both in vitro and in vivo have been independently linked to AD pathogeneses. In particular, BIN1 positively regulates the transcription of critical AD-related genes (including *Apoe*, *Trem2*, and *Tyropb*) under homeostatic conditions. This regulatory interaction between BIN1 and other AD-associated genes illustrates the complexities of multi-aetiological disorders and the challenge of pinpointing specific genetic variations which account for the entire spectrum of pathologies associated with AD. However, BIN1’s involvement in both homeostasis- and inflammation-specific signal regulation suggests that BIN1 may act as a central regulator of microglial activation status, with implications for mediating the kaleidoscope of DAM transcriptional profiles. BIN1’s known function in membrane remodelling and endocytic trafficking is consistent with such a broad role in regulating phenotypic changes. Indeed, this is exemplified by our findings that BIN1 regulates surface expression of CD11c in response to LPS administration, independent of *Itgax* transcript levels (assayed in from the same FACS-isolated cell samples). Unravelling the potential co-operative role(s) played by BIN1 and microglial surface receptors in DAM transformation presents a crucial area for future investigations. However, BIN1’s relationship with surface receptors appears more complex than the singular function of endocytosis or endosome recycling, as exemplified by our findings of CX3CR1 dysregulation.

Despite some limitations due to the loss of one functional *Cx3cr1* allele, by utilising the *Cx3cr1*^Cre^ driver line, we fortuitously found that BIN1 counteracts changes elicited by *Cx3cr1* haploinsufficiency, demonstrating BIN1’s involvement with another central regulator of microglial function. Compared with *Bin1*^fl/fl^ mice having normal *Cx3cr1* expression, *Cx3cr1*^CreER^ mice exhibited an exaggerated response to systemic LPS challenge, consistent with prior observations [96]. As *Cx3cr1* is downregulated during Stage I DAM transition [25], the microglia in the *Cx3cr1*^CreER^ driver line may be primed towards DAM transition. When *Bin1* was deleted in *Cx3cr1*^CreER^ mice, we observed an attenuation of the effect caused by *Cx3cr1* haploinsufficiency. This finding suggests that BIN1 may serve as a mediator of CX3CR1 receptor activation in the CX3CL1 (fractalkine)-CX3CR1 signaling axis. This conclusion is supported by the microglial cell population data showing a significant LPS-induced increase of DAM CD11c^+^ (CD11b^+^CD45^int^) in *Cx3cr1*^CreER^ mice, which is abrogated following the deletion of *Bin1* alleles in microglia. Our in vivo transcriptional dataset revealed *Bin1* to regulate gene transcripts in a manner inverse to *Cx3cr1*. Ongoing investigations of microglia-specific BIN1 deletion using the *Tmem119*^Cre-ERT2^ driver [97] in models of AD pathologies will help determine whether microglial BIN1 may function to protect against both amyloid and tau aggregations.

## Conclusions

Our study demonstrates that BIN1 regulates crucial elements of inflammatory response in microglia. Importantly, key characteristics of microglial phenotypic transition, including the upregulation of *Ifitm3* and surface expression of CD11c, are BIN1-dependent. We conclude that BIN1 expression is central for appropriate microglial responses to CNS challenge, and that AD risk may arise through impaired BIN1 functionality in microglia.

## Supplementary Information

**Additional file 1: Fig. S1.** BIN1 expression in mouse and human microglia from transcriptomic and proteomic datasets. **(A-B)** BIN1 transcript and protein levels in brain cells reported in large-scale datasets. **(A)** Transcript abundances from purified neural cells were described by Zhang et al. [16]. OPC, oligodendrocyte precursor cells; NF Oli, newly-formed oligodendrocytes; Myel Oli, myelinating oligodendrocytes; End, endothelial cells. **(B)** Comparison of protein abundance data (log2 transformed abundance values from label-free quantitative studies) from purified mouse neural cell types [20]. **(C)** A comparison of microglial protein levels of BIN1 (percentile rank abundance) between humans (aged post-mortem human brain-derived microglia) and several mouse models. The plotted data was compiled from several independent quantitative mass spectrometry datasets: CD11b + magnetic-activated cell sorting (MACS) from 6 to 7-mo-old female C57BL6J mice that received vehicle or LPS (4 daily i.p. doses), or a transgenic mouse model of AD pathology (5XFAD), CD11b + microglia from 3-mo-old C57BL6J mice purified by MACS, or fluorescent activated cell sorting (FACS), and immortalised microglial BV2 cells (untreated or LPS-treated for 24 h) [26, 98, 99] **(D)** Singe nucleus RNA sequencing data demonstrates the high level of BIN1 transcripts found in microglia and oligodendrocytes in fresh-frozen post-mortem human brain tissue [100]. (**E)** RNA sequencing mRNA abundance (log2 transformed) from isolated human microglia across different age groups shows high-level BIN1 mRNA abundance in microglia across the life span in the human brain [101].

**Additional file 2: Fig. S2.** Exon 11 splicing in microglia. **(A)** RT-PCR across exon 11 found no inclusion of exon 11 in FACS-isolated microglia from mouse brain, with no change in splicing following LPS injections (left). iMG cells differentiated from human iPSCs showed negligible inclusion of exon 11 (right). Some low-level inclusion of exon 11 is evident in the grey matter sample of human post-mortem brain tissue. **(B)** Schematic of BIN1 exons and primer locations for RT-PCR amplification across exon 7, exon 11, and the CLAP domain / exon 17. The strategy used to discern alternate splicing of exons 13-17 is indicated. The four mouse microglial Bin1 isoforms identified in this study are depicted at the bottom. BAR, the BIN-amphiphysin/Rvs domain; PI, the phosphoinositide binding motif (encoded by the muscle-specific exon 11), CLAP, the clathrin and AP2 binding domain, SH3, the Src homology 3 domain.

**Additional file 3: Fig. S3.** *Bin1* siRNA treatment of primary microglia dysregulates DAM gene transcripts without affecting cell viability. CD11b + enriched primary mouse microglia (p0-3) were cultured for 48 h in the presence of either sham siRNA or Bin1 siRNA (equimolar concentrations). **(A)** Cell viability within the CD45+ microglia population was assessed by flow cytometry (Live/Dead Fixable Blue viability dye). *N* = 3 independent experiments were performed per condition. **(B)** Bin1 transcript levels (NanoString) increase following LPS stimulation of cultured microglia. **(C)** Volcano plot showing key genes differentially expressed following Bin1 KD. **(D)** Transcript expression of key AD-related microglial genes are dysregulated following the loss of Bin1 expression, an effect augmented by LPS-induced inflammatory signaling.

**Additional file 4: Fig. S4.** Transcript cluster analysis of in vitro dataset demonstrates the extent of transcriptional dysregulation by *Bin1* KD in primary cultured microglia. **(A)** Volcano plot of transcript expression following LPS exposure identifies several AD- and DAM-related genes affected by this endotoxin. The color scheme depicted is based on microglial gene co-expression module assignment, as described in Rangaraju et al. [21]. **(B)** Volcano plot illustrating genes regulated by BIN1 following LPS exposure highlights several inflammatory genes dysregulated by Bin1 siRNA. **(C-F)** Gene ontology expression analysis identified important cellular functions affected by misexpressed gene clusters.

**Additional file 5: Fig. S5.** Flow cytometry gating strategy for mouse brain-isolated microglia, weight, and temperature analysis during LPS injections. **(A)** Cells isolated from mouse brains were gated by scatter (single mononuclear cells) and fluorescence (CD11b^+^CD45^int^) to sort microglial populations. **(B-C)** LPS-injected mice lost significant weight during injections. Reduced Cx3cr1 expression (in Cx3cr1^CreER^) augmented the LPS-induced weight-loss recorded in control (Bin1^fl/fl^) animals, which was attenuated by the additional deletion of microglial Bin1 (Cx3cr1^CreER^-Bin1 cKO). **(D-E)** LPS had no effect on body temperature before or after injection. **(F)** Immunofluorescence staining of Bin1^fl/fl^ mouse brains suggests that peripheral LPS injections did not affect the microglial expression or localization of BIN1. **(G)** NanoString analysis of mRNA transcripts found no change in Bin1 expression following LPS injections. **(H)** Relative levels of Bin1 iso10 mRNA transcripts quantified from FACS-isolated microglia, following saline or LPS injections.

**Additional file 6: Fig. S6.** Deletion of microglial *Bin1* does not impact cell morphology. **(A)** Following the deletion of *Bin* from microglia (*Cx3cr1*^CreER^-*Bin1* cKO), no obvious change in microglial morphology was observed. There didn’t seem to be any effect on LPS administration. **(B)** Quantification of FracLac hull and circle morphometric analysis of microglia from LPS-injected mice. Microglia in the primary somatosensory cortex (SSp) of *Cx3cr1*^CreER^-*Bin1* cKO mice had larger span ratios than *Cx3cr1*^CreER^ control mice. *, *p* < 0.05; by Mann-Whitney U test. **(C)** Representative images of data presented in B. All data plotted as mean ± SEM.

**Additional file 7: Fig. S7.** Summary of in vivo transcriptional changes and cytokine production. **(A-B)** Genes which are positively (A) and negatively (B) regulated by BIN1 (i.e., decrease and increase respectively with Bin1 knockout) are summarised from the in vivo NanoString dataset by raw transcript counts (top panel) and normalized expression (relative to Bin1^fl/fl^ as the WT equivalent; bottom panel). **(C)** Summary of cytokine expression in brain lysates (measured by Luminex). Data plotted as mean *±* SEM.

**Additional file 8: Fig. S8.** Whole-brain qRT-PCR analysis of selected homeostatic- and DAM-related genes, and master myeloid-regulating transcription factor. Raw dataset for the analysis summarised in Fig. 8A. Transcript levels of genes encoding homeostatic proteins (P2ry12, Tmem119), DAM proteins (Itgax, Cst7, Cd68, Trem2, Spp1), and transcription factor (Sfpi1) were analysed by qRT-PCR. With the exception of the DAM-related gene Spp1, all were upregulated following LPS stimulation in a BIN1-dependent manner. *, *p* < 0.05; **, *p* < 0.01; by unpaired t-test. Data plotted as mean ± SEM. *, *p* < 0.05.

**Additional file 9: Fig. S9.** Generation of BV2 KO microglia lacking BIN1 expression by CRISPR/Cas9 gene editing. **(A)** Lentiviral constructs expressing a sgRNA targeting a region within the *Bin1* invariant exon 3 (KO) or a non-target sgRNA were used to generate stably transduced pools of BV2 KO and control (WT) cells. Two independent pools of WT and KO were further characterized. Sequencing (using a reverse primer) across the target sequence of the two KO pools as well as the sequences of individual cloned inserts from the PCR products are aligned to *Bin1* exon 3 sequence. Numbering is based on the RefSeq NM_009668.2. **(B)** WT control and *Bin1* KO pools retain similar morphology. **(C)** Immunoblot analysis demonstrates that stable *Bin1* KO BV2 pools do not express BIN1 protein under basal conditions or following LPS stimulation.

**Additional file 10: Supplementary Tables S1-6.**

## Data Availability

All data generated and analysed during this study are included in this published article and its supplementary information files.

## Abbreviations

Aβ: Amyloid-β
AD: Alzheimer’s disease
ANOVA: Analysis of variance
BAR: BIN/amphiphysin/Rvs binding region
BIN1: Bridging integrator 1
cKO: Conditional knockout
CLAP: Clathrin-associated protein binding region
DAM: Disease-associated microglia
FACS: Fluorescence-activated cell sorting
FDR: False discovery rate
iPSC: Induced pluripotent stem cells
iMG: Induced microglial-like cell
IRFs: Interferon regulatory factors
Iso: Isoform
KD: Knockdown
LOAD: Late-onset Alzheimer’s disease
LPS: Lipopolysaccharide
MACS: Magnetic-activated cell sorting
MAGMA: Multi-marker analysis of genomic annotation
PC: Principal component
SH3: SRC homology 3 domain
siRNA: Small interfering RNA
tSNE: T-distributed stochastic neighbor embedding
WT: Wild-type
