# Supplementary material for: BIN1 is a key regulator of proinflammatory and neurodegeneration-related activation in microglia
Source: Mol Neurodegener. 2022 May 7;17:33. doi: 10.1186/s13024-022-00535-x (PMC9077874; doi:10.1186/s13024-022-00535-x)
Supplement: Supplementary file 1 — Additional file 1: Fig. S1. BIN1 expression in mouse and human microglia from transcriptomic and proteomic datasets. (A-B) BIN1 transcript and protein levels in brain cells reported in large-scale datasets. (A) Transcript abundances from purified neural cells were described by Zhang et al. [16]. OPC, oligodendrocyte precursor cells; NF Oli, newly-formed oligodendrocytes; Myel Oli, myelinating oligodendrocytes; End, endothelial cells. (B) Comparison of protein abundance data (log2 transformed abundance values from label-free quantitative studies) from purified mouse neural cell types [20]. (C) A comparison of microglial protein levels of BIN1 (percentile rank abundance) between humans (aged post-mortem human brain-derived microglia) and several mouse models. The plotted data was compiled from several independent quantitative mass spectrometry datasets: CD11b + magnetic-activated cell sorting (MACS) from 6 to 7-mo-old female C57BL6J mice that received vehicle or LPS (4 daily i.p. doses), or a transgenic mouse model of AD pathology (5XFAD), CD11b + microglia from 3-mo-old C57BL6J mice purified by MACS, or fluorescent activated cell sorting (FACS), and immortalised microglial BV2 cells (untreated or LPS-treated for 24 h) [26, 98, 99] (D) Singe nucleus RNA sequencing data demonstrates the high level of BIN1 transcripts found in microglia and oligodendrocytes in fresh-frozen post-mortem human brain tissue [100]. (E) RNA sequencing mRNA abundance (log2 transformed) from isolated human microglia across different age groups shows high-level BIN1 mRNA abundance in microglia across the life span in the human brain [101]. [file 13024_2022_535_MOESM1_ESM.pdf]

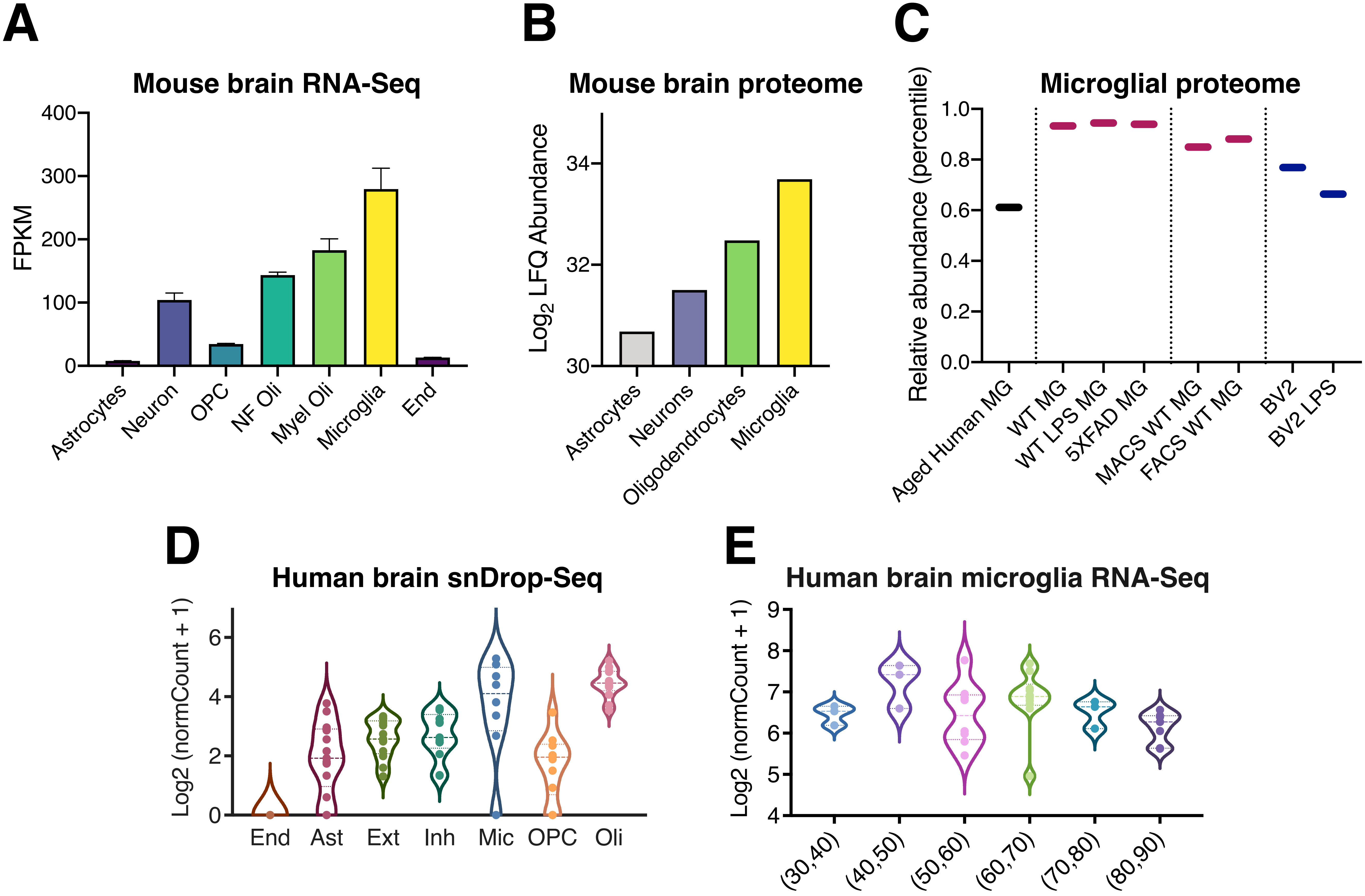

A
Mouse brain RNA-Seq
FPKM
400
300
200
100
0
Astrocytes
Neuron
OPC
NF Oli
Myel Oli
Microglia
End
B
Mouse brain proteome
Log2 LFQ Abundance
34
32
30
Astrocytes
Neurons
Oligodendrocytes
Microglia
C
Microglial proteome
Relative abundance (percentile)
1.0
0.8
0.6
0.4
0.2
0.0
Aged Human MG
WT MG
WT LPS MG
5XFAD MG
MACS WT MG
FACS WT MG
BV2
BV2 LPS
D
Human brain snDrop-Seq
Log2 (normCount + 1)
6
4
2
0
End
Ast
Ext
Inh
Mic
OPC
Oli
E
Human brain microglia RNA-Seq
Log2 (normCount + 1)
9
8
7
6
5
4
(30,40)
(40,50)
(50,60)
(60,70)
(70,80)
(80,90)
