# Supplementary figures and images for: BIN1 is a key regulator of proinflammatory and neurodegeneration-related activation in microglia
Source: Mol Neurodegener. 2022 May 7;17:33. doi: 10.1186/s13024-022-00535-x (PMC9077874; doi:10.1186/s13024-022-00535-x)

**A**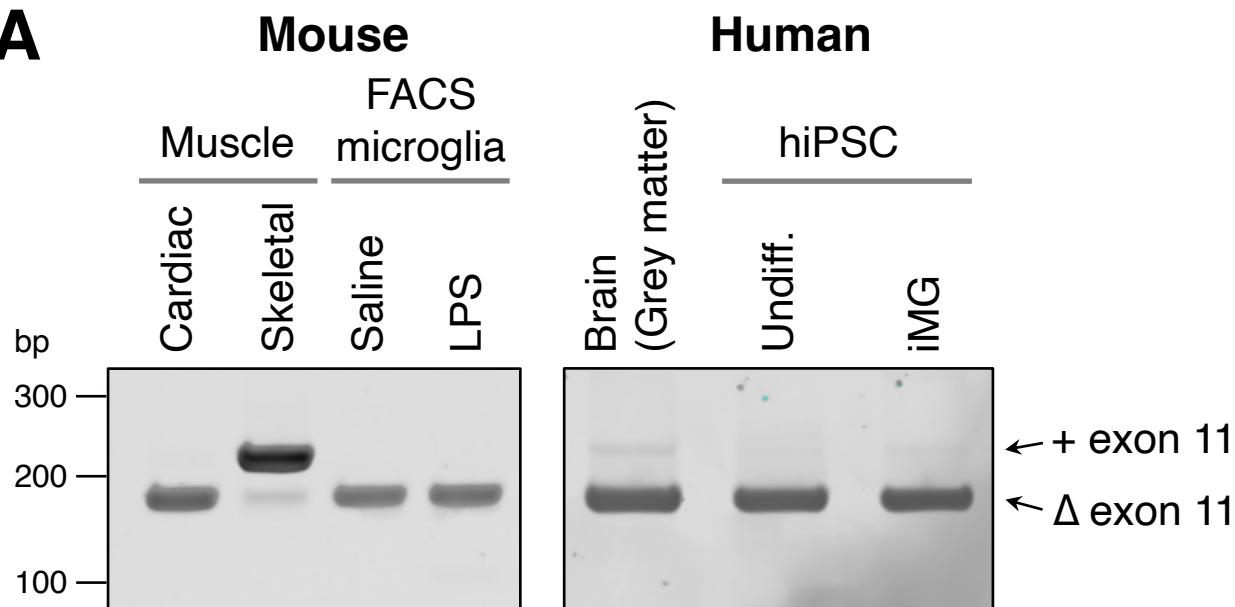**B**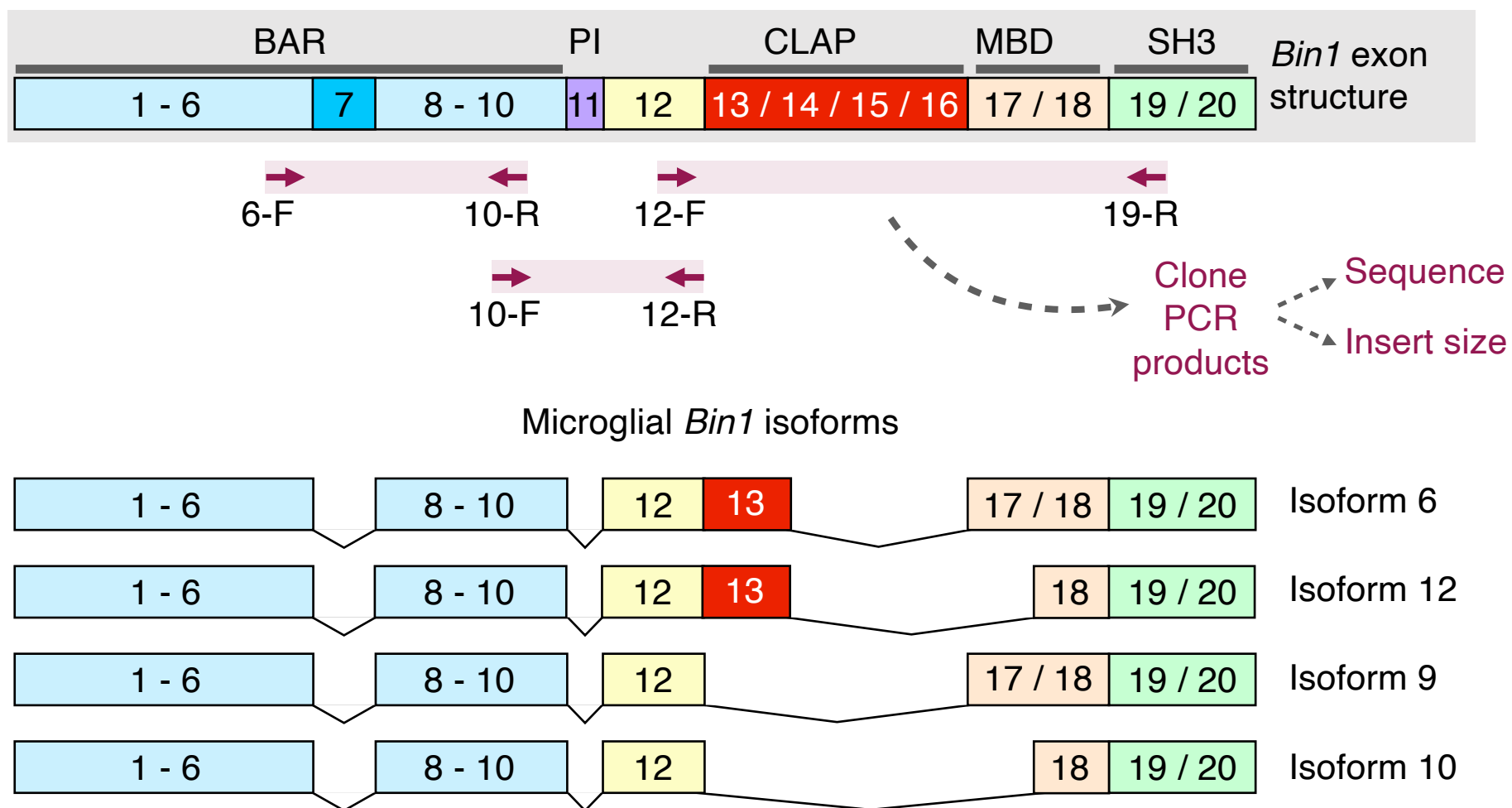

Supplement: Supplementary file 2 — Additional file 2: Fig. S2. Exon 11 splicing in microglia. (A) RT-PCR across exon 11 found no inclusion of exon 11 in FACS-isolated microglia from mouse brain, with no change in splicing following LPS injections (left). iMG cells differentiated from human iPSCs showed negligible inclusion of exon 11 (right). Some low-level inclusion of exon 11 is evident in the grey matter sample of human post-mortem brain tissue. (B) Schematic of BIN1 exons and primer locations for RT-PCR amplification across exon 7, exon 11, and the CLAP domain / exon 17. The strategy used to discern alternate splicing of exons 13-17 is indicated. The four mouse microglial Bin1 isoforms identified in this study are depicted at the bottom. BAR, the BIN-amphiphysin/Rvs domain; PI, the phosphoinositide binding motif (encoded by the muscle-specific exon 11), CLAP, the clathrin and AP2 binding domain, SH3, the Src homology 3 domain. [file 13024_2022_535_MOESM2_ESM.pdf]

**A**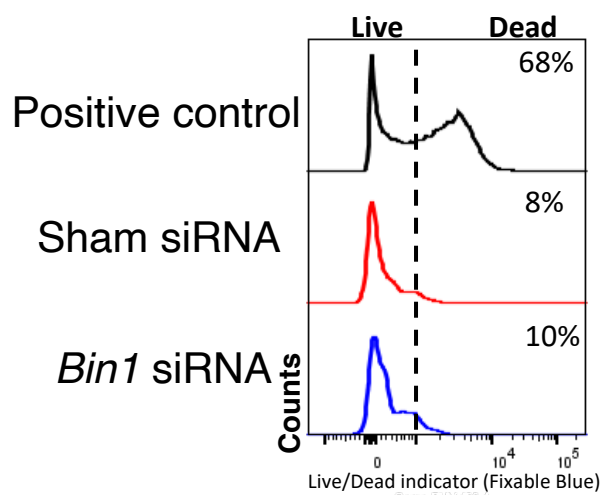**B**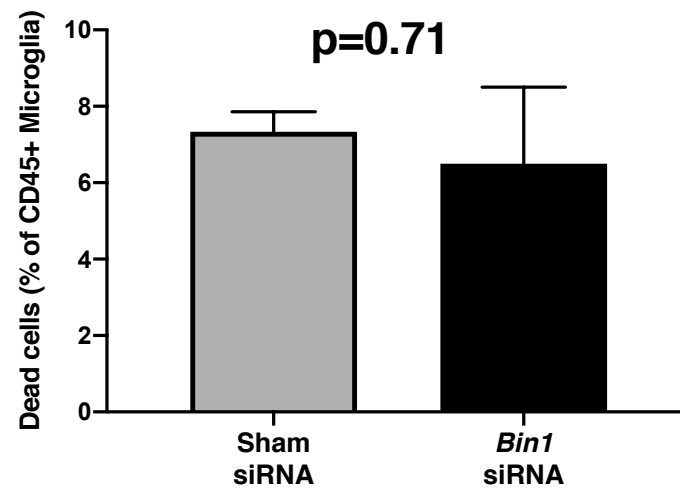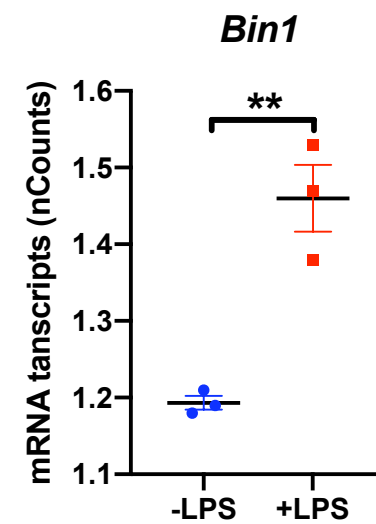**C**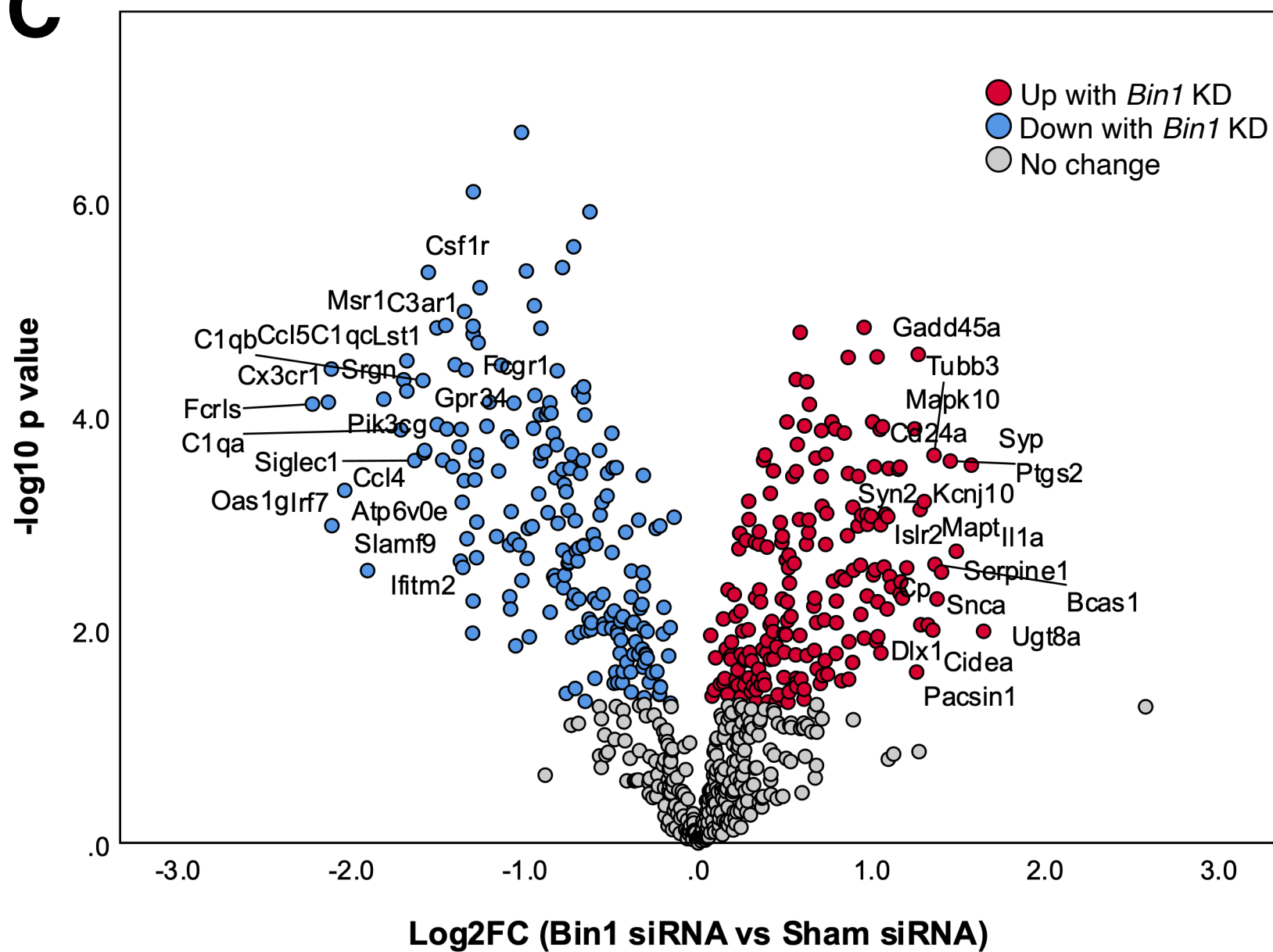**D**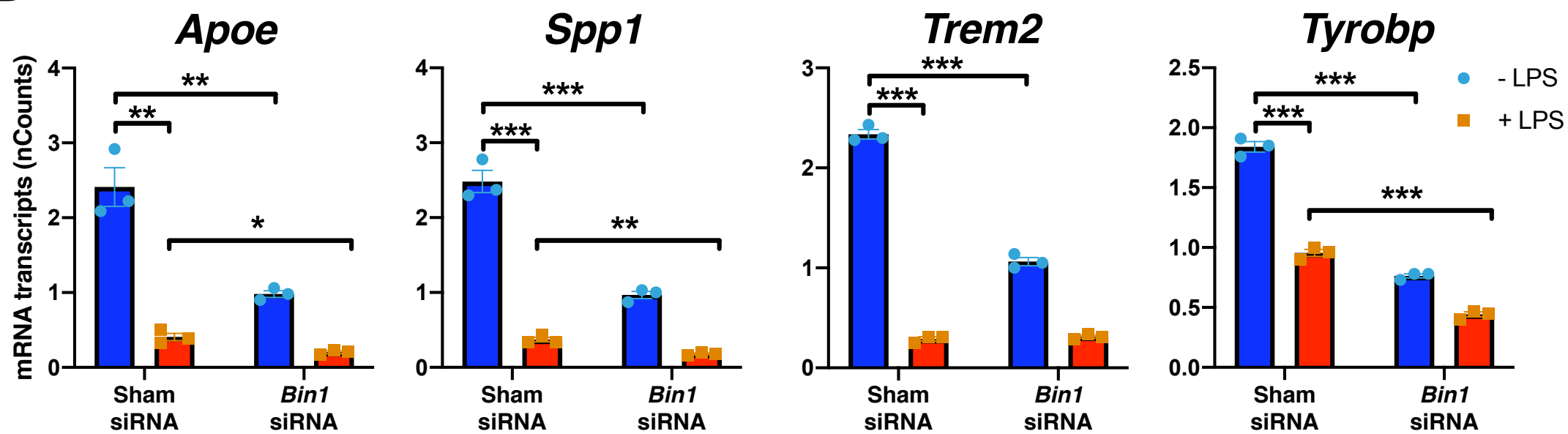

Supplement: Supplementary file 3 — Additional file 3: Fig. S3. Bin1 siRNA treatment of primary microglia dysregulates DAM gene transcripts without affecting cell viability. CD11b + enriched primary mouse microglia (p0-3) were cultured for 48 h in the presence of either sham siRNA or Bin1 siRNA (equimolar concentrations). (A) Cell viability within the CD45+ microglia population was assessed by flow cytometry (Live/Dead Fixable Blue viability dye). N = 3 independent experiments were performed per condition. (B) Bin1 transcript levels (NanoString) increase following LPS stimulation of cultured microglia. (C) Volcano plot showing key genes differentially expressed following Bin1 KD. (D) Transcript expression of key AD-related microglial genes are dysregulated following the loss of Bin1 expression, an effect augmented by LPS-induced inflammatory signaling. [file 13024_2022_535_MOESM3_ESM.pdf]

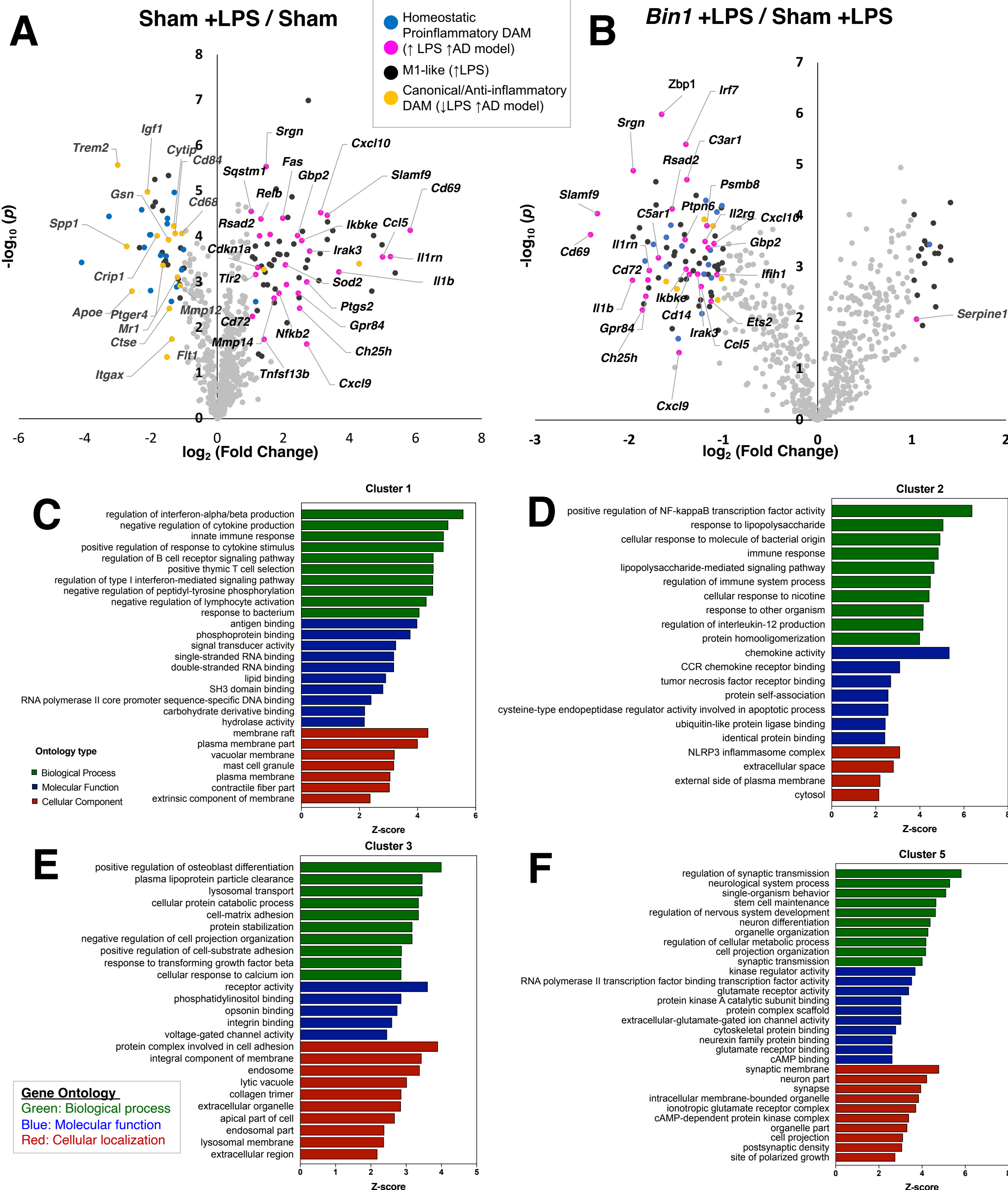

Supplement: Supplementary file 4 — Additional file 4: Fig. S4. Transcript cluster analysis of in vitro dataset demonstrates the extent of transcriptional dysregulation by Bin1 KD in primary cultured microglia. (A) Volcano plot of transcript expression following LPS exposure identifies several AD- and DAM-related genes affected by this endotoxin. The color scheme depicted is based on microglial gene co-expression module assignment, as described in Rangaraju et al. [21]. (B) Volcano plot illustrating genes regulated by BIN1 following LPS exposure highlights several inflammatory genes dysregulated by Bin1 siRNA. (C-F) Gene ontology expression analysis identified important cellular functions affected by misexpressed gene clusters. [file 13024_2022_535_MOESM4_ESM.pdf]

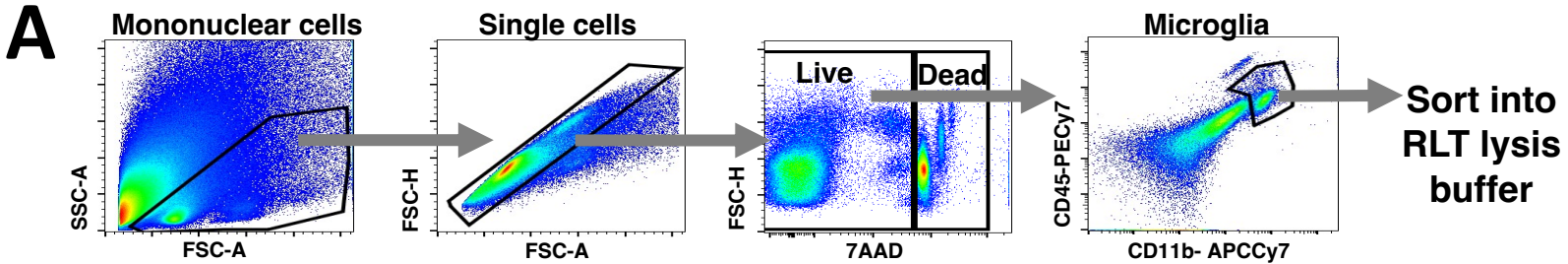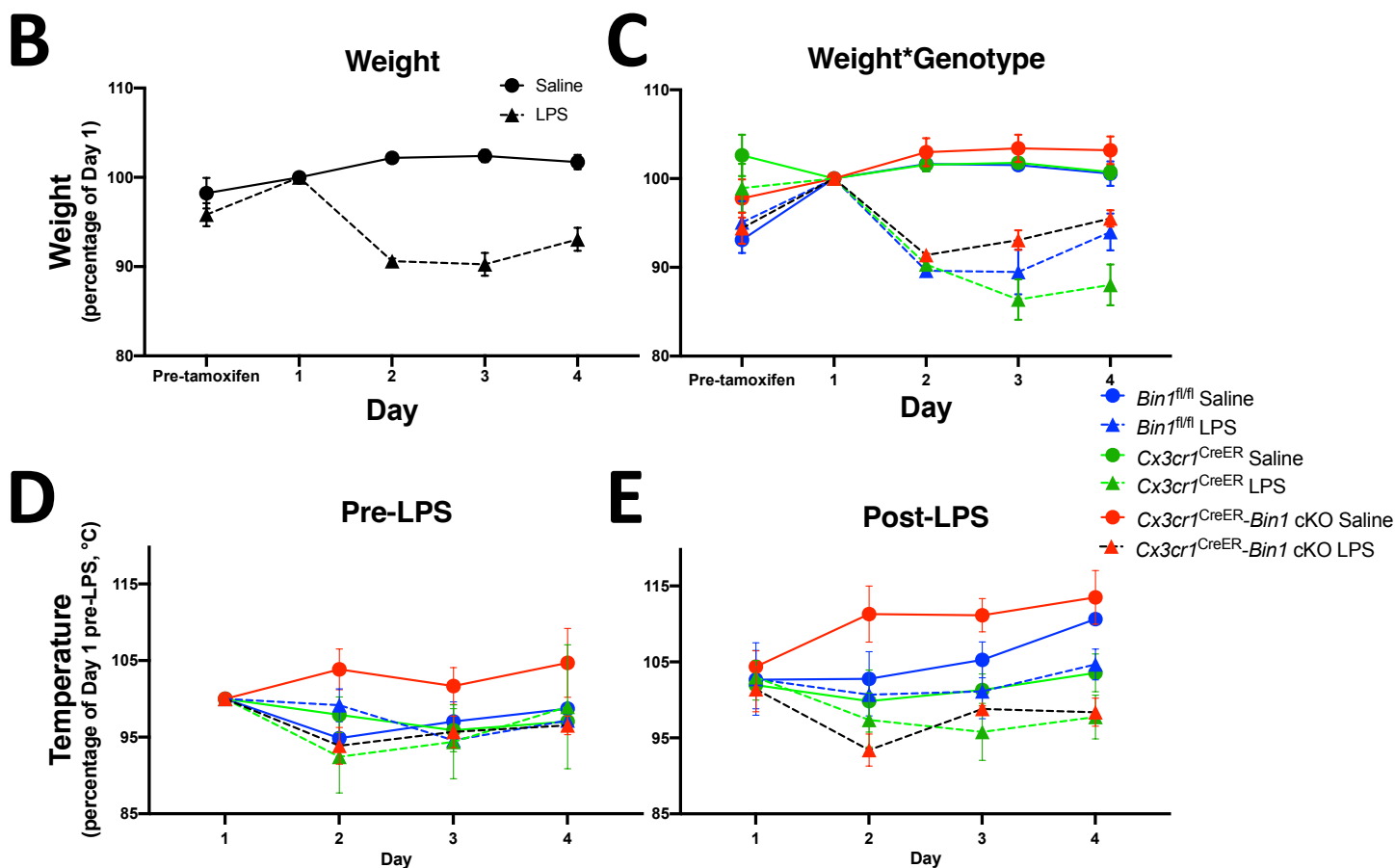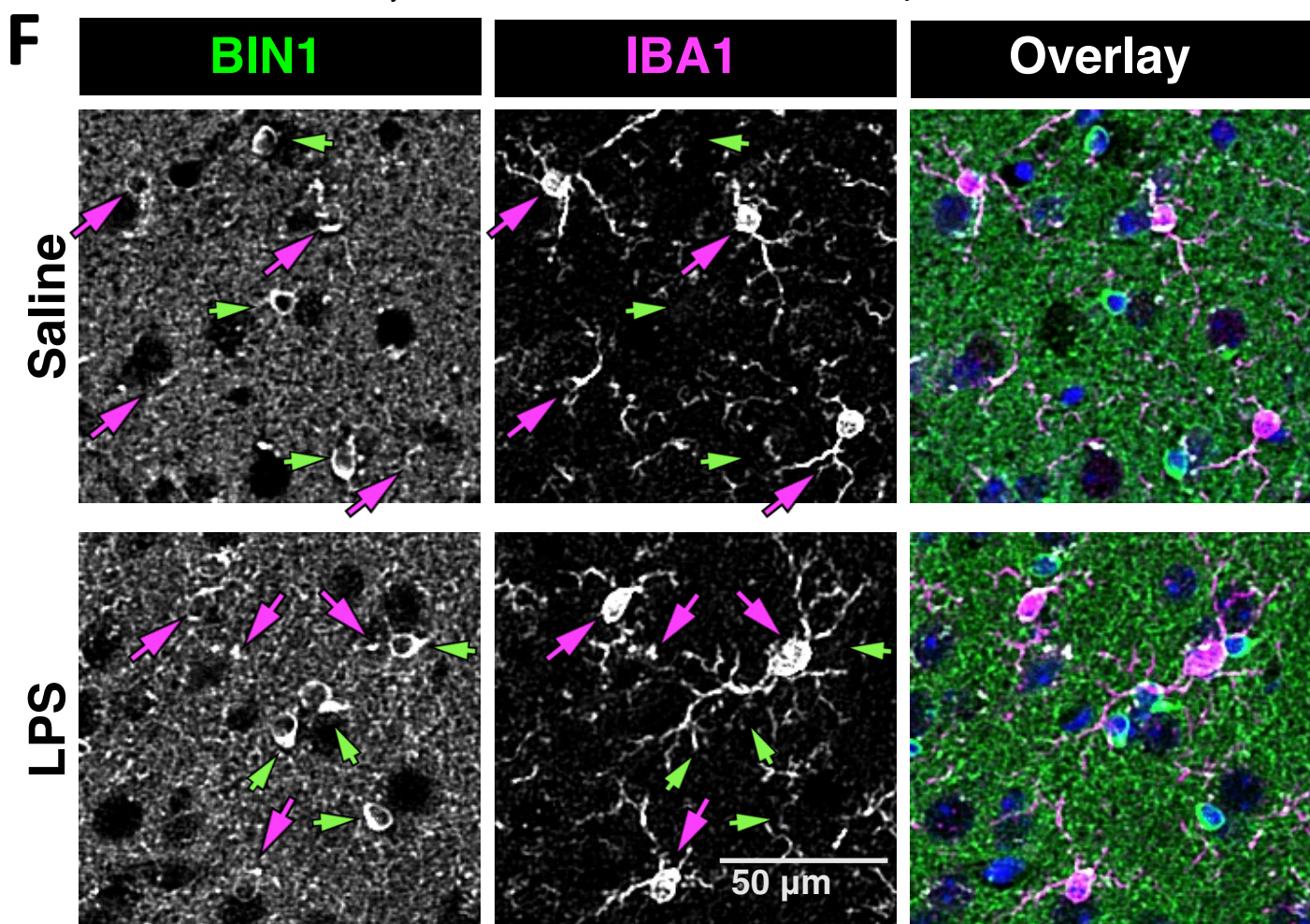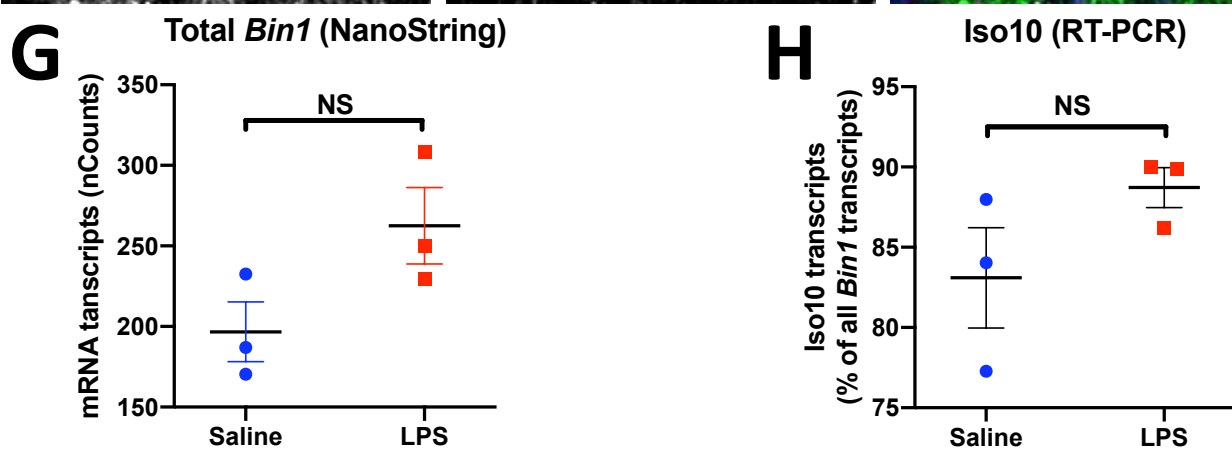

Supplement: Supplementary file 5 — Additional file 5: Fig. S5. Flow cytometry gating strategy for mouse brain-isolated microglia, weight, and temperature analysis during LPS injections. (A) Cells isolated from mouse brains were gated by scatter (single mononuclear cells) and fluorescence (CD11b+CD45int) to sort microglial populations. (B-C) LPS-injected mice lost significant weight during injections. Reduced Cx3cr1 expression (in Cx3cr1CreER) augmented the LPS-induced weight-loss recorded in control (Bin1fl/fl) animals, which was attenuated by the additional deletion of microglial Bin1 (Cx3cr1CreER-Bin1 cKO). (D-E) LPS had no effect on body temperature before or after injection. (F) Immunofluorescence staining of Bin1fl/fl mouse brains suggests that peripheral LPS injections did not affect the microglial expression or localization of BIN1. (G) NanoString analysis of mRNA transcripts found no change in Bin1 expression following LPS injections. (H) Relative levels of Bin1 iso10 mRNA transcripts quantified from FACS-isolated microglia, following saline or LPS injections. [file 13024_2022_535_MOESM5_ESM.pdf]

**A**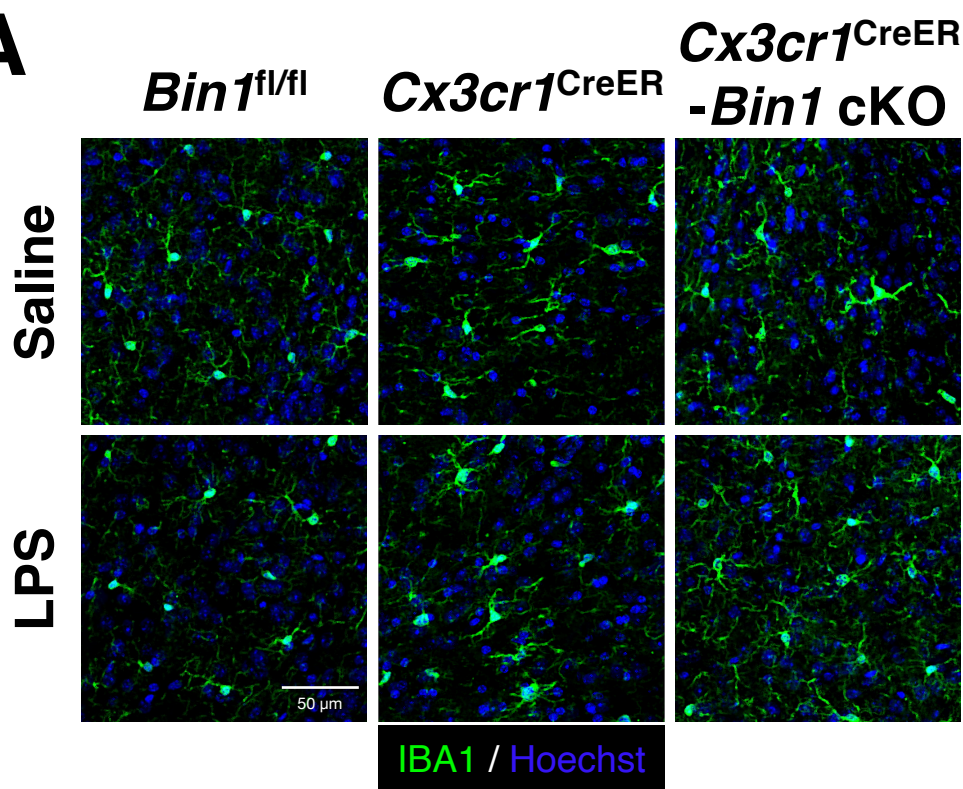**B**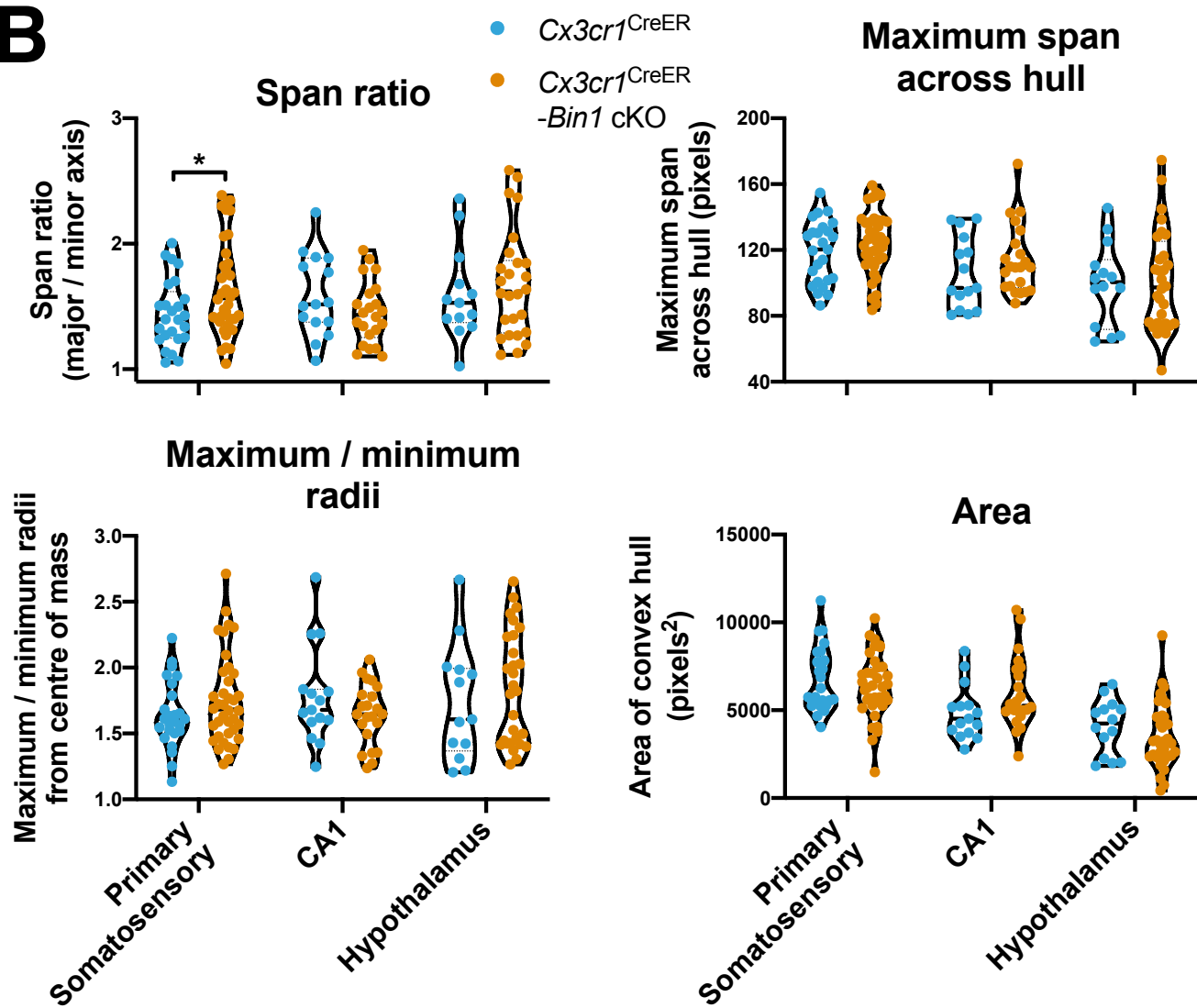**C**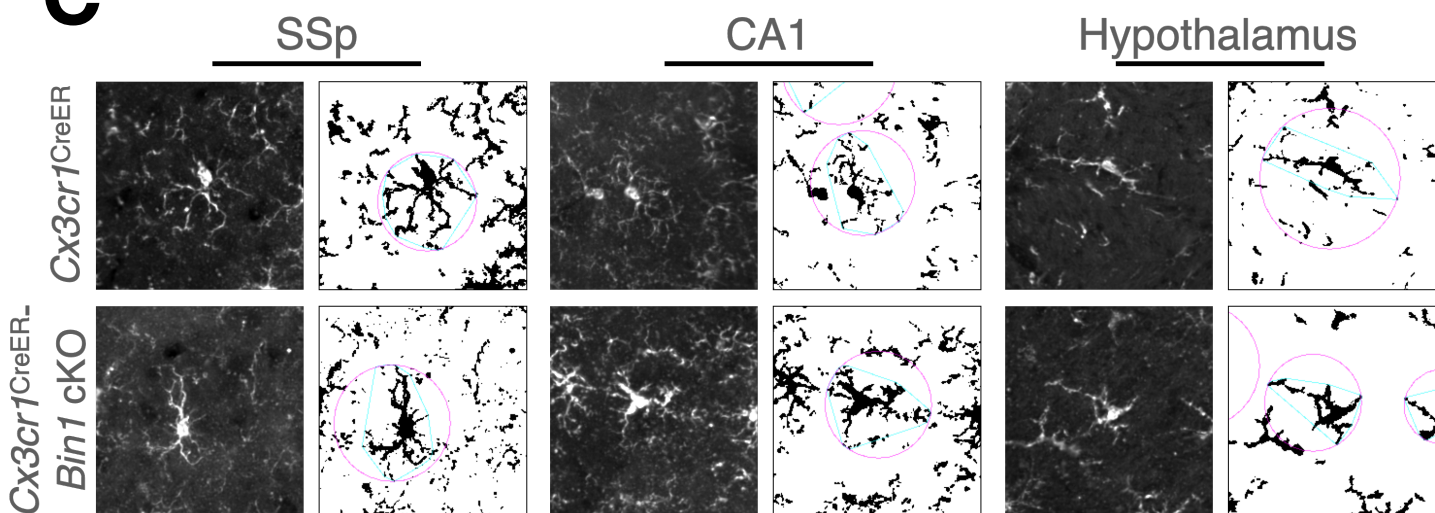

Supplement: Supplementary file 6 — Additional file 6: Fig. S6. Deletion of microglial Bin1 does not impact cell morphology. (A) Following the deletion of Bin from microglia (Cx3cr1CreER-Bin1 cKO), no obvious change in microglial morphology was observed. There didn’t seem to be any effect on LPS administration. (B) Quantification of FracLac hull and circle morphometric analysis of microglia from LPS-injected mice. Microglia in the primary somatosensory cortex (SSp) of Cx3cr1CreER-Bin1 cKO mice had larger span ratios than Cx3cr1CreER control mice. *, p < 0.05; by Mann-Whitney U test. (C) Representative images of data presented in B. All data plotted as mean ± SEM. [file 13024_2022_535_MOESM6_ESM.pdf]

# A

## BIN1 negatively regulated

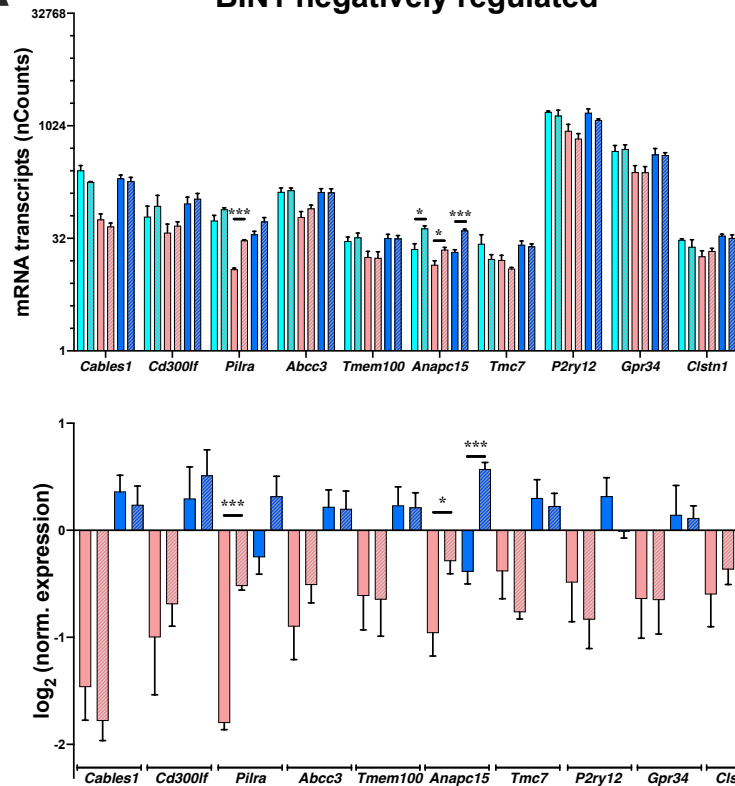

# B

## BIN1 positively regulated

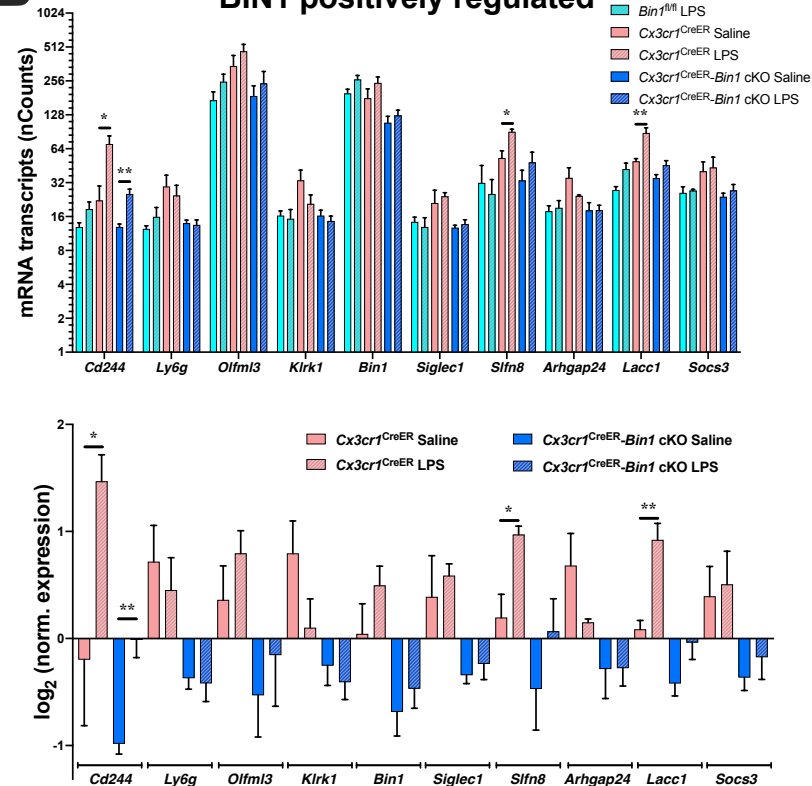

# C

## Luminex (brain lysate)

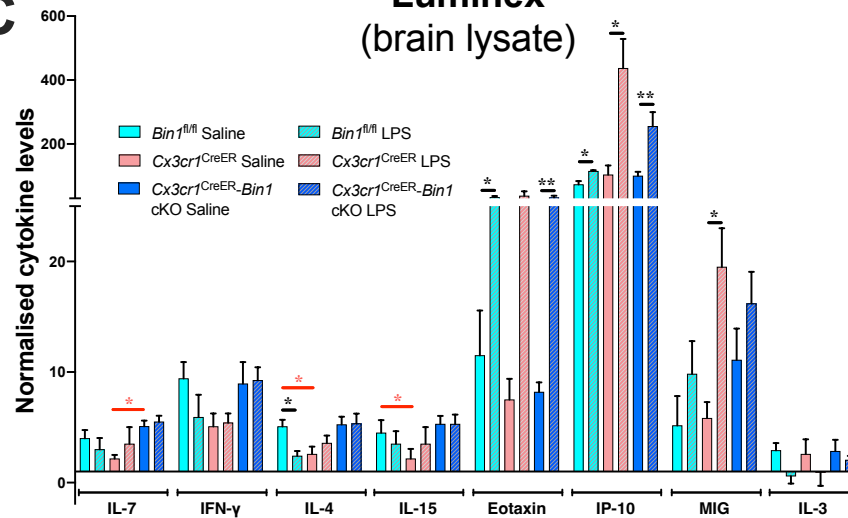

Supplement: Supplementary file 7 — Additional file 7: Fig. S7. Summary of in vivo transcriptional changes and cytokine production. (A-B) Genes which are positively (A) and negatively (B) regulated by BIN1 (i.e., decrease and increase respectively with Bin1 knockout) are summarised from the in vivo NanoString dataset by raw transcript counts (top panel) and normalized expression (relative to Bin1fl/fl as the WT equivalent; bottom panel). (C) Summary of cytokine expression in brain lysates (measured by Luminex). Data plotted as mean ± SEM. [file 13024_2022_535_MOESM7_ESM.pdf]

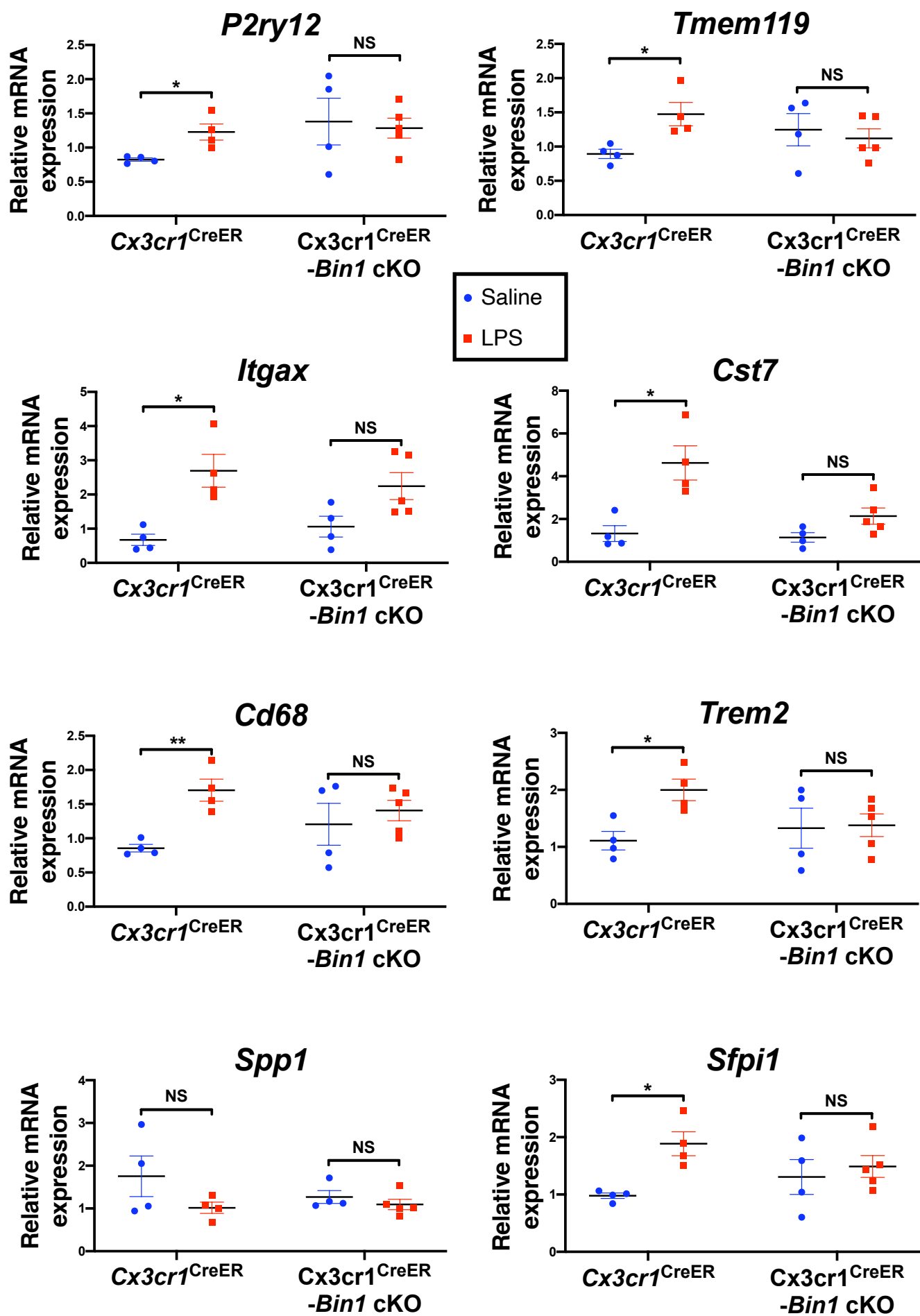

Supplement: Supplementary file 8 — Additional file 8: Fig. S8. Whole-brain qRT-PCR analysis of selected homeostatic- and DAM-related genes, and master myeloid-regulating transcription factor. Raw dataset for the analysis summarised in Fig. 8A. Transcript levels of genes encoding homeostatic proteins (P2ry12, Tmem119), DAM proteins (Itgax, Cst7, Cd68, Trem2, Spp1), and transcription factor (Sfpi1) were analysed by qRT-PCR. With the exception of the DAM-related gene Spp1, all were upregulated following LPS stimulation in a BIN1-dependent manner. *, p < 0.05; **, p < 0.01; by unpaired t-test. Data plotted as mean ± SEM. *, p < 0.05. [file 13024_2022_535_MOESM8_ESM.pdf]
