# Supplementary material for: BIN1 is a key regulator of proinflammatory and neurodegeneration-related activation in microglia
Source: Mol Neurodegener. 2022 May 7;17:33. doi: 10.1186/s13024-022-00535-x (PMC9077874; doi:10.1186/s13024-022-00535-x)
Supplement: Supplementary file 9 — Additional file 9: Fig. S9. Generation of BV2 KO microglia lacking BIN1 expression by CRISPR/Cas9 gene editing. (A) Lentiviral constructs expressing a sgRNA targeting a region within the Bin1 invariant exon 3 (KO) or a non-target sgRNA were used to generate stably transduced pools of BV2 KO and control (WT) cells. Two independent pools of WT and KO were further characterized. Sequencing (using a reverse primer) across the target sequence of the two KO pools as well as the sequences of individual cloned inserts from the PCR products are aligned to Bin1 exon 3 sequence. Numbering is based on the RefSeq NM_009668.2. (B) WT control and Bin1 KO pools retain similar morphology. (C) Immunoblot analysis demonstrates that stable Bin1 KO BV2 pools do not express BIN1 protein under basal conditions or following LPS stimulation. [file 13024_2022_535_MOESM9_ESM.pdf]

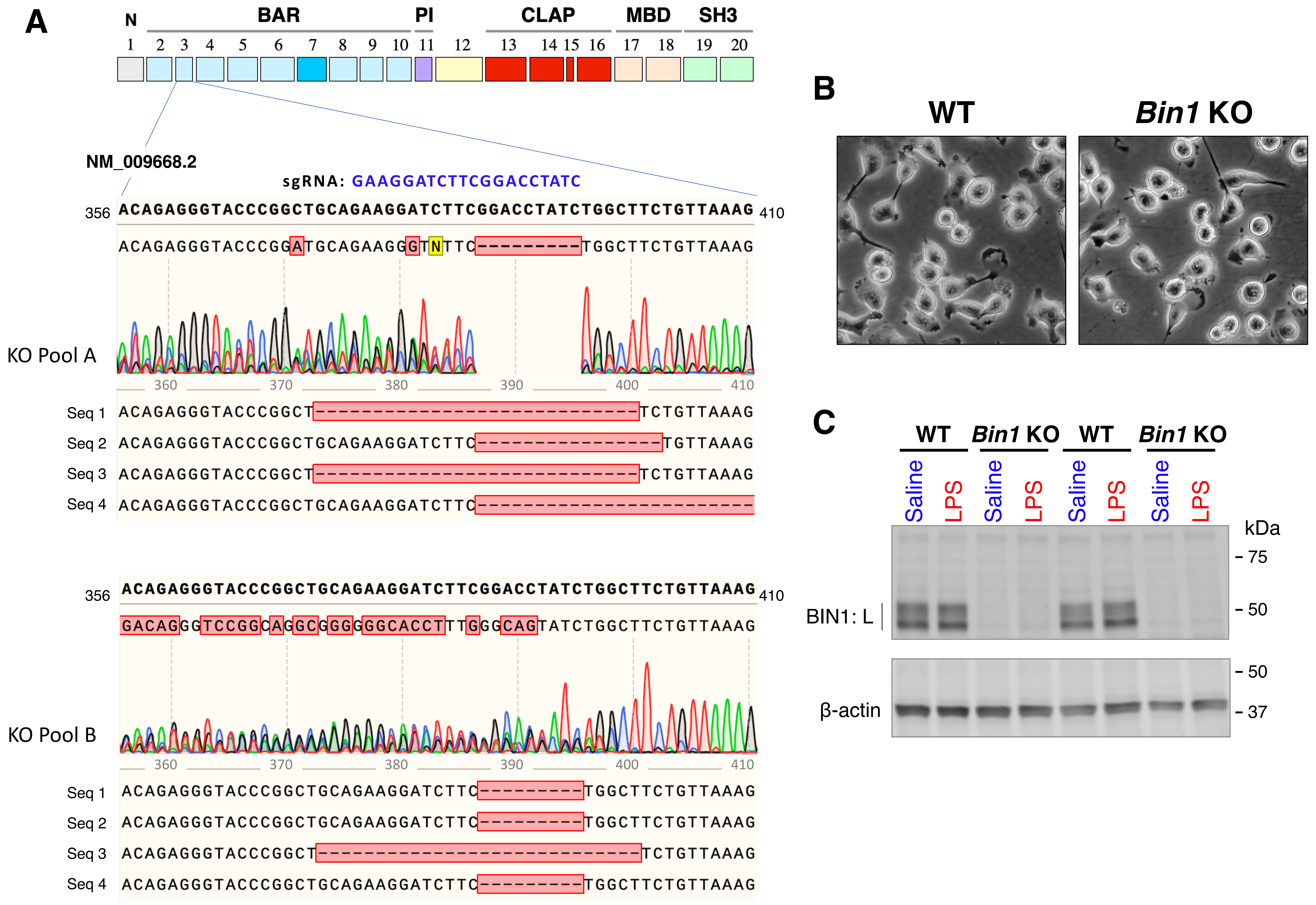
A
N
BAR
PI
CLAP
MBD
SH3
1 2 3 4 5 6 7 8 9 10 11 12 13 14 15 16 17 18 19 20
NM_009668.2
sgRNA: GAAGGATCTTCGGACCTATC
356 ACAGAGGGTACCCGGCTGCAGAAGGATCTTCGGACCTATCTGGCTTCTGTTAAAG 410
ACAGAGGGTACCCGGATGCAGAAGGGTNTTC---------TGGCTTCTGTTAAAG
KO Pool A
360 370 380 390 400 410
Seq 1 ACAGAGGGTACCCGGCT----------------------------TCTGTTAAAG
Seq 2 ACAGAGGGTACCCGGCTGCAGAAGGATCTTC----------------TGTTAAAG
Seq 3 ACAGAGGGTACCCGGCT----------------------------TCTGTTAAAG
Seq 4 ACAGAGGGTACCCGGCTGCAGAAGGATCTTC------------------------
356 ACAGAGGGTACCCGGCTGCAGAAGGATCTTCGGACCTATCTGGCTTCTGTTAAAG 410
GACAGGGTCCGGCAGGCGGGGGCACCTTTGGGCAGTATCTGGCTTCTGTTAAAG
KO Pool B
360 370 380 390 400 410
Seq 1 ACAGAGGGTACCCGGCTGCAGAAGGATCTTC---------TGGCTTCTGTTAAAG
Seq 2 ACAGAGGGTACCCGGCTGCAGAAGGATCTTC---------TGGCTTCTGTTAAAG
Seq 3 ACAGAGGGTACCCGGCT----------------------------TCTGTTAAAG
Seq 4 ACAGAGGGTACCCGGCTGCAGAAGGATCTTC---------TGGCTTCTGTTAAAG
B
WT
Bin1 KO
C
WT
Bin1 KO
WT
Bin1 KO
Saline
LPS
Saline
LPS
Saline
LPS
Saline
LPS
kDa
75
50
BIN1: L
50
β-actin
37
